# The cohesin loader SCC2 contains a PHD finger that is required for meiosis in land plants

**DOI:** 10.1371/journal.pgen.1008849

**Published:** 2020-06-09

**Authors:** Hongkuan Wang, Wanyue Xu, Yujin Sun, Qichao Lian, Cong Wang, Chaoyi Yu, Chengpeng He, Jun Wang, Hong Ma, Gregory P. Copenhaver, Yingxiang Wang

**Affiliations:** 1 State Key Laboratory of Genetic Engineering and Ministry of Education Key Laboratory of Biodiversity Sciences and Ecological Engineering, Institute of Plant Biology, School of Life Sciences, Fudan University, Shanghai, China; 2 Center for Epigenetics, Van Andel Institute, Grand Rapids, Michigan, United States of America; 3 Department of Biology and the Integrative Program for Biological and Genome Sciences, University of North Carolina at Chapel Hill, Chapel Hill, North Carolina, United States of America; 4 Department of Biology, the Huck Institutes of the Life Sciences, the Pennsylvania State University, University Park, Pennsylvania, United States of America; 5 Lineberger Comprehensive Cancer Center, University of North Carolina School of Medicine, Chapel Hill, North Carolina, United States of America; HudsonAlpha Institute for Biotechnology, UNITED STATES

## Abstract

Cohesin, a multisubunit protein complex, is required for holding sister chromatids together during mitosis and meiosis. The recruitment of cohesin by the sister chromatid cohesion 2/4 (SCC2/4) complex has been extensively studied in *Saccharomyces cerevisiae* mitosis, but its role in mitosis and meiosis remains poorly understood in multicellular organisms, because complete loss-of-function of either gene causes embryonic lethality. Here, we identified a weak allele of *Atscc2* (*Atscc2-5*) that has only minor defects in vegetative development but exhibits a significant reduction in fertility. Cytological analyses of *Atscc2-5* reveal multiple meiotic phenotypes including defects in chromosomal axis formation, meiosis-specific cohesin loading, homolog pairing and synapsis, and *AtSPO11-1*-dependent double strand break repair. Surprisingly, even though AtSCC2 interacts with AtSCC4 *in vitro* and *in vivo*, meiosis-specific knockdown of *AtSCC4* expression does not cause any meiotic defect, suggesting that the SCC2-SCC4 complex has divergent roles in mitosis and meiosis. SCC2 homologs from land plants have a unique plant homeodomain (PHD) motif not found in other species. We show that the AtSCC2 PHD domain can bind to the N terminus of histones and is required for meiosis but not mitosis. Taken together, our results provide evidence that unlike SCC2 in other organisms, SCC2 requires a functional PHD domain during meiosis in land plants.

## Author summary

Cohesin is required to hold sister chromatids together during mitosis and meiosis. The recruitment of cohesin, mediated by the sister chromatid cohesion 2/4 (SCC2/4) complex, has been extensively studied in yeast mitosis. Because complete loss-of-function of either gene causes embryonic lethality in multicellular organisms, its role in mitosis and meiosis remains poorly understood. Here, we show that *Arabidopsis* SCC2 functions in meiosis in an AtSCC4-independent manner. We also demonstrate that SCC2 in land plants has a PHD domain not found in animal or fungal homologs and is critical for meiotic function but not mitosis.

## Introduction

The faithful transmission of chromosomes to daughter cells is an essential feature of the cell cycle in most eukaryotes. Improper chromosome segregation during mitosis or meiosis leads to aneuploidy, which in turn can cause defects in growth, development and reproduction [1, 2]. During meiosis, to ensure the proper segregation of chromosomes, the cohesin complex holds sister chromatids together from the end of S phase until anaphase II [3].

Cohesin is a member of the ancient and conserved SMC (Structural Maintenance of Chromosomes) protein family [3]. In *S*. *cerevisiae*, the core cohesin complex consists of Smc1, Smc3, Scc3 and an α-kleisin (Scc1 and Rec8 in mitosis and meiosis respectively) [4]. Smc1 interacts with Smc3 via their hinge domains, and their ATPase head domains are linked by the kleisin subunit [5]. Scc3 is able to interact with the kleisin subunit [6]. These four subunits form a ring-shaped structure [7]. *Arabidopsis* has single copies of *AtSMC1*/*AtTTN8*, *AtSMC3*/*AtTTN7* and *AtSCC3* [8–11]. However, in *Arabidopsis*, there are four kleisin proteins: SYN1/DIF1/REC8, SYN2/RAD21.1, SYN3/RAD21.2, and SYN4/RAD21.3 [10, 12–14]. Among them, AtSYN3 is required for male and female meiosis and may have functions other than as a subunit of cohesin, including a role in regulating nucleolar structure [10, 15]. AtSYN1, the orthologue of yeast Rec8, is a meiosis-specific cohesin subunit and is essential for chromosome condensation, sister chromatid cohesion, double strand break (DSB) repair and mono-orientation of meiotic chromosome [9, 16, 17]. Other accessory proteins such as AtSWITCH1 (AtSWI1) and AtWAPL1/AtWAPL2, also help mediate cohesin association or disassociation with chromatin [18–21].

Cohesin is recruited onto chromosomes by the conserved heterodimeric SCC2-SCC4 complex in most model organisms [21–25]. The SCC2 C terminus contains several HEAT repeats that are required for forming a hook-like structure, which is critical for loading cohesin onto DNA [26]. The N-terminal end of SCC2 can interact with SCC4 to form a globular head domain [26]. *Arabidopsis* SCC4 is a small, 726 amino acid protein with a predicted tetratricopeptide repeat (TPR) 12 domain [27]. In *S*. *cerevisiae* mitosis, Scc4 may stabilize Scc2 *in vivo* and facilitates cohesin loading at centromeres [26, 28, 29]. In addition, *in vitro* experiments with the C-terminal end of human Scc2 showed it can interact specifically with the HsSmc1-HsSmc3 heterodimer, but HsScc4 does not bind to cohesin [24]. Loading assays using *Schizosaccharomyces pombe in vitro* reconstituted cohesin complexes indicated that Mis4^Scc2^ is sufficient for cohesin loading onto DNA, in the absence of Ssl3^Scc4^ [30]. Recent biochemical and genetic analyses in *S*. *cerevisiae* also support the idea that Scc2 is sufficient for stimulating cohesin’s ATPase activity in the absence of Scc4 [31]. Furthermore, the mechanisms that recruit SCC4 to specific chromatin sites have been reported in several species. In *S*. *cerevisiae*, Scc4 can be directly recruited to centromeres by the phosphorylated kinetochore protein Ctf19 [29]. In *Xenopus*, a complex of Scc4 and the N terminus of Scc2 is sufficient to bind chromatin, through interacting with pre-replication complex (pre-RC), but cannot recruit cohesin [32, 33]. The human SCC2-SCC4 complex also interacts with the MCM2-7 complex [34]. In *Zea mays* (maize), SCC4/Dek15 interacts with several chromatin remodeling proteins [35]. Together, these results suggest that recruitment of SCC2/4 onto chromatin likely depends on SCC4-interacting factors such as Ctf19 and the MCM2-7 complex, while SCC2 is important for loading cohesin.

*Arabidopsis* has single copy of *SCC2* and homozygous full loss-of-function alleles are embryonic lethal [36]. Plants with RNAi-induced knock-down of *AtSCC2* exhibit reduced fertility and meiotic defects in pairing of homologous chromosomes, chromosomal axis formation and sister chromatid cohesion [36]. Interestingly, unlike other taxa, SCC2 orthologs from plants contain a PHD domain [36]. However, the function of the SCC2 PHD domain in mitosis and meiosis has not been investigated. Recently, *SCC4* was also identified in *Arabidopsis* and maize. Similar to *AtSCC2*, complete loss of function of *SCC4* is also embryonic lethal in both *Arabidopsis* and maize, consistent with their roles in mitotic cell division [27, 35, 37]. Nonetheless, the gene expression pattern of *AtSCC2* does not fully overlap with *AtSCC4* [27]. In addition, they have non-overlapping roles in process such as endosperm development [27, 36].

We recovered a mutant in a screen for male sterility and identified the locus as *AtSCC2*. The non-lethal *Atscc2* mutant (designated *Atscc2-5*) provided an opportunity to study its meiotic function. Consistent with previous *AtSCC2* RNAi knock-down phenotypes, *Atscc2-5* has meiotic defects in chromosomal axis formation, pairing of homologous chromosomes, synapsis and recombination. Our analyses demonstrate that *AtSCC2* acts in the same pathway as *AtSYN1* and *AtWAPL1/2*, and participates in *AtSPO11-1*-depedent DSB repair. We also provide evidence that the N-terminal end of AtSCC2 interacts with AtSCC4 both *in vitro* and *in vivo*, and meiosis-specific knockdown of *AtSCC4* does not cause any meiotic defects, suggesting that the role of the SCC2-SCC4 complex is divergent in mitosis and meiosis. Using *in vitro* binding assays we also show that the plant-specific *AtSCC2* PHD domain binds to the N terminus of H2A, H3 and H4. Further *in vivo* functional analyses demonstrate that the PHD domain of AtSCC2 is required for meiotic function, but not for vegetative growth, which adds to our mechanistic understanding of cohesins in plants.

## Results

### Identification of a hypomorphic *Atscc2* allele

To better understand the genetic mechanisms controlling meiosis, we screened an EMS mutagenized population and identified a sterile plant (*line88*). After backcrossing *line88* with wild type (WT) Col-0 for three generations, the stable *line88* plants have a slight vegetative growth defect at four and eight-week-old stages compared to WT (S1A and S1B Fig). However, *line88* plants are almost completely sterile and produce very few seeds (0.7 ± 0.8 seeds per silique, n = 21, p-value = 4.9E-48, two-tailed Student’s *t* test) compared to WT (55.5 ± 2.5 seeds per silique, n = 21) (S1C and S1D Fig). WT stamens are plump, and their mature stigmas are covered with pollen (S1E Fig), while *line88* plants lack pollen on their stigmas suggesting a defect in male gamete production (S1F Fig). Alexander staining for pollen viability showed a significant reduction in the number of viable pollen grains per anther in *line88* (average = 20.3 ± 12.4, n = 19, p-value = 2.1E-31, two-tailed Student’s *t* test), compared to WT (average = 581.1 ± 58.3, n = 19) (S1H and S1G Fig). Consistent with these observations, toluidine blue staining of tetrad-stage microspores showed that 56.1% (n = 57) of male meioses in *line88* plants result in polyads with variable sized microspores (S1J Fig), while WT plants produce only tetrads with four similarly sized microspores (S1I Fig, n = 48). These phenotypes suggest a defect in meiotic chromosome segregation in *line88*.

Heterozygous F1 plants (*line88* as the female parent and WT *Lansberg erecta* (L*er*) as the male parent) have normal fertility, indicating that the mutation is recessive. We allowed the F1 plants to self-fertilize and used bulked-segregant analysis of 97 sterile F2 plants to map the mutant locus to a region on the upper arm of chromosome 5 (S2A and S2B Fig). The region includes four candidate genes, including *AT5G15540* (*AtSCC2*) which contains a mutation compared to the WT reference sequence. The single nucleotide polymorphism is a G to A transition in the C terminus of *AtSCC2* (5,049,797 bp) that does not result in an amino acid change, but is predicted to disrupt the splice site at the 3' end of exon 22 (S2C Fig). Genotyping the alleles in the F2 progeny shows that the ratio of homozygous WT (n = 409) and heterozygotes (n = 816) is consistent with a 1:2 ratio (p(χ^2^) = 0.99, chi-square test), whereas the ratio of mutant (n = 278) and WT phenotypes (n = 1225) deviates significantly from a 1:3 ratio (S1 Table) (χ^2^ = 33.6 > χ^2^_0.05_ = 3.8, chi-square test). These segregation ratios are consistent with a single recessive allele with an incompletely penetrant embryonic lethal phenotype.

**Fig 1:**
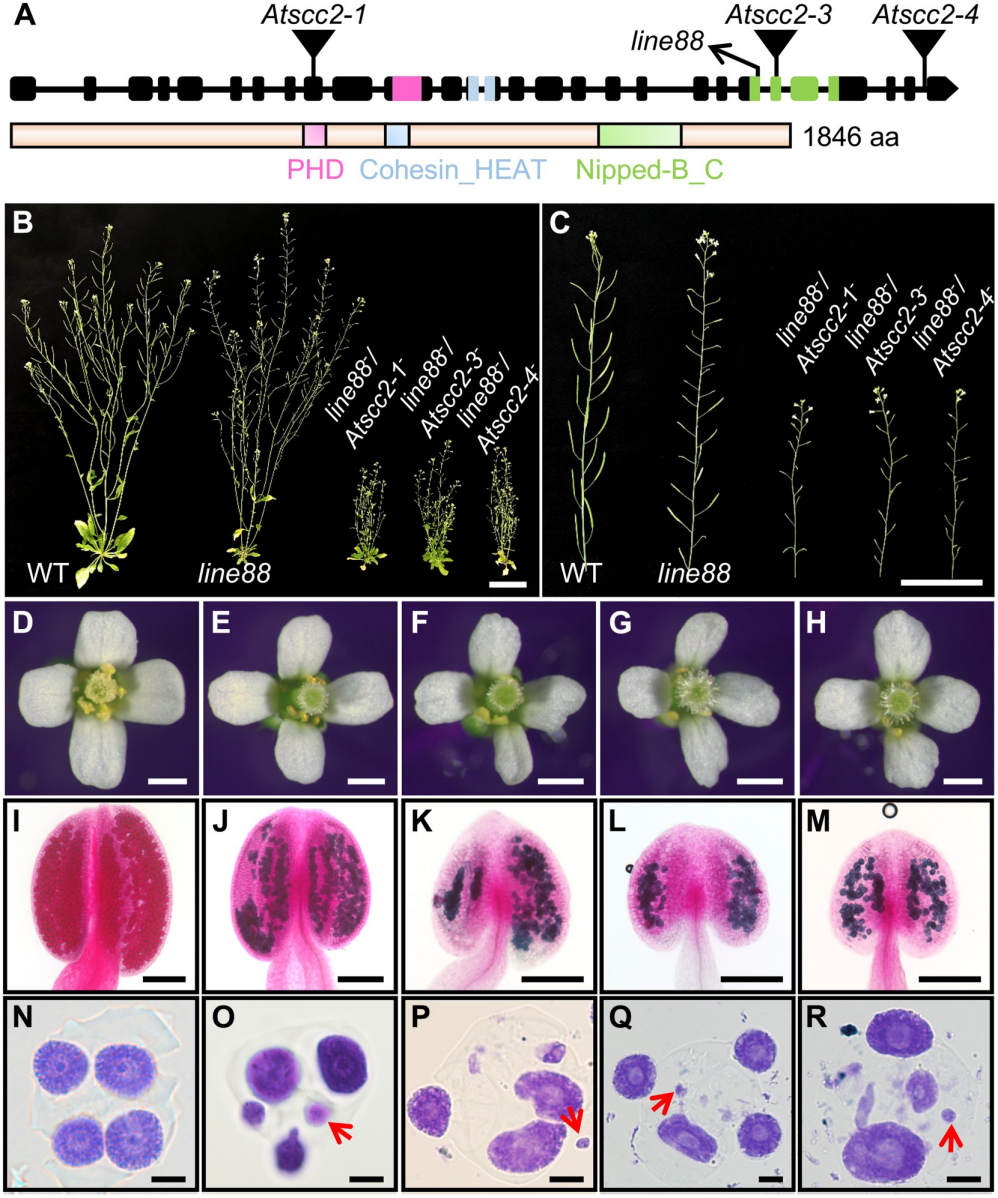
Identification of *Atscc2-5*. (A) A diagram of *AtSCC2* gene and AtSCC2 protein structure. Mutant alleles are marked above the gene structure (inverted triangles and diagonal arrow). (B) WT, *line88* and three compound heterozygous mutants of *line88* with individual *Atscc2* alleles. Bar = 3 cm. (C) Primary stems of WT, *line88* and three compound heterozygous plants. Bar = 3 cm. (D-H) Open flowers of WT, *line88* and three compound heterozygous plants. Bar = 0.5 mm. (I-M) Alexander staining of WT, *line88* and three compound heterozygous plant anthers. Bar = 100 μm. (N-R) Toluidine blue staining of tetrad-stage microspores of WT, *line88* and three compound heterozygous plants. Red arrows indicate the abnormal micronucleus in tetrad stage. Bar = 5 μm.

Previous studies showed that complete loss-of-function of *AtSCC2* leads to embryonic lethality [36]. To confirm that the *line88* phenotype is caused by the mutation we mapped to *AtSCC2*, we crossed *line88* with heterozygotes of three T-DNA alleles of *AtSCC2* (*Atscc2-1*, *Atscc2-3* and *Atscc2-4*) (Fig 1A and 1B). These three T-DNA alleles are embryonic lethal as homozygotes as shown by the 1:2 segregation ratio of homozygous WT plants and heterozygotes in the F2 progeny of *Atscc2-1*^*+/-*^ (67/110, p(χ^2^) = 0.23, chi-square test), *Atscc2-3*^*+/-*^ (33/63, p(χ^2^) = 0.91, chi-square test) and *Atscc2-4*^*+/-*^ (33/58, p(χ^2^) = 0.63, chi-square test) plants (S1 Table), and the complete lack of homozygous mutant F2 (S1 Table). Consistently, the ratio of compound heterozygous F1 plants of two independent alleles (*line88*^-^*/Atscc2-1*^-^ and *line88*^-^/*Atscc2-3*^-^) with their corresponding *Atscc2-5*^*+/-*^ heterozygous F1 plants is 1:1 (χ^2^ ≤ χ_0.05_^2^ = 3.84, chi-square test) (S2 Table). The ratio of F1 compound heterozygous of *line88*^-^/*Atscc2-4*^-^ with *Atscc2-5*^*+/-*^ heterozygous F1 plants is not consistent with 1:1 (χ^2^ = 4.02 > χ_0.05_^2^ = 3.84), probably due to low population numbers or an incompletely penetrant embryonic lethal phenotype. All of the compound heterozygous F1 progeny had severe vegetative growth defects compared with the homozygous *line88* plants and WT (Fig 1B), supporting the essential role of *AtSCC2* in mitosis. These compound heterozygous plants also have short siliques and no pollen on their stigmas (Fig 1C and 1F–1H) compared with WT (Fig 1C and 1D), similar to *line88* (Fig 1C and 1E). Pollen viability in the compound heterozygous plants is reduced (Fig 1J–1M), compared with WT (Fig 1I). The compound heterozygotes also have abnormal tetrad-stage microspores with polyads of uneven size (Fig 1P–1R), similar to *line88* (Fig 1O) and unlike WT (Fig 1N). These results demonstrate that *line88* is a mutant allele of *AtSCC2* which we will henceforth call *Atscc2-5*.

We also used trans-complementation with the WT AtSCC2 coding sequence fused to a FLAG-tag and expressed by the *AtACTIN7* promoter to confirm that the *Atscc2-5* phenotypes are caused by a mutation in the *AtSCC2* locus. The *AtACT7*::*AtSCC2-FLAG* transgene is able to rescue the fertility, pollen viability and meiotic defects of *Atscc2-5*^-/-^ plants (S3A–S3G Fig). Western blotting with anti-FLAG antibody confirmed that the AtSCC2-FLAG fusion is expressed at the expected size in the transgenic plants (211.6 kD; S3E Fig). These results validate the point mutation in *AtSCC2* as the causative lesion for the mutant phenotypes we observe in *Atscc2-5*.

The *Atscc2-5* point mutation at the border of the exon 22 (S2C and S4A Figs) is predicted to disrupt a splice site. To test this hypothesis, we amplified the *AtSCC2* transcript using primers spanning the point mutation and found that *Atscc2-5* expresses two transcripts: one WT *AtSCC2* transcript and one 47 bp deletion version (S4B Fig). Quantitative real-time reverse transcription PCR (qRT-PCR) demonstrated that the full length *AtSCC2* transcript is significantly lower (~5% in leaves and ~2% in meiocytes) in *Atscc2-5* compared to WT (S4C Fig). This is also supported by RNA-seq data from WT and *Atscc2-5* meiocytes (S5A Fig). We speculate that the reduced level of full length *AtSCC2* transcript in *Atscc2-5* may be caused by a premature termination codon inducing nonsense-mediated mRNA decay (NMD) [38]. Disruption of the splice site appears to trigger the use of an upstream cryptic splice site, resulting in a 47 bp deletion in the mRNA which creates a premature stop codon and may yield a truncated AtSCC2 protein (1–1400 amino acids) in *Atscc2-5* (S5B Fig). We speculate that the residual intact *AtSCC2* transcripts in *Atscc2-5* are sufficient to rescue embryonic lethality, but still cause aberrant meiotic phenotypes.

### The *Atscc2-5* mutant shows multiple meiotic defects

**Fig 2:**
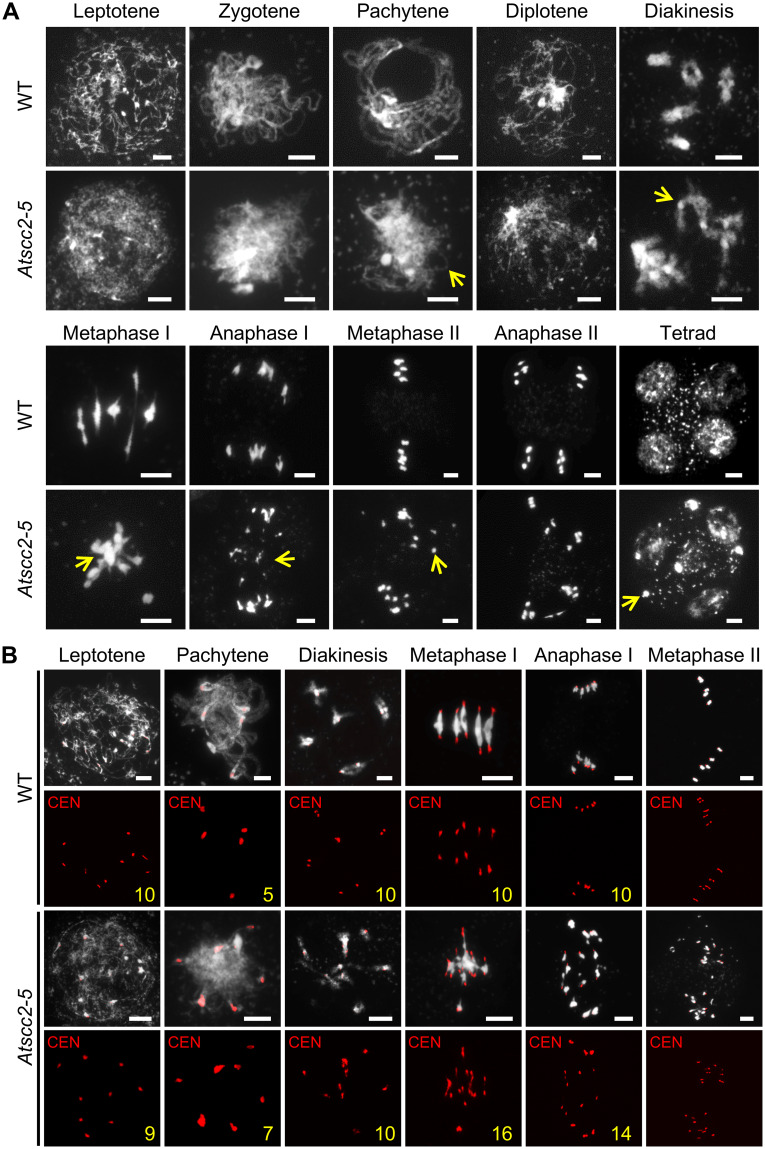
Chromosome morphology of *Atscc2-5* and WT male meiocytes. (A) Chromosome spreads of WT and *Atscc2-5* male meiocytes stained by DAPI. Yellow arrows indicate the asynaptic chromosomes, univalent, abnormal chromosomal entanglements or fragments in *Atscc2-5*. Bar = 5 μm. (B) Fluorescence *in situ* hybridization (FISH) of WT and *Atscc2-5* chromosomes using a centromere probe. Yellow numbers indicate the number of centromeres in the meiocytes. Bar = 5 μm.

We stained chromosome spreads from WT and mutant pollen mother cells (PMCs) with 4’, 6-diamidino-2-phenylindole (DAPI) to investigate the meiotic defects in *Atscc2-5* (Fig 2A). At leptotene, the *Atscc2-5* chromosomes are very rough and appear less condensed compared to WT which appear as distinct thin threads. At zygotene and pachytene, WT chromosomes continue to condense, homologs align, and synapsis results in thick thread-like structures. In *Atscc2-5* meiocytes, chromosome at similar stages remain relatively thin and defuse, indicating a defect in synapsis. Following desynapsis at diplotene, WT homologs remain associated through chiasmata at crossover sites and form five highly condensed bivalents at diakinesis. In contrast, condensation of *Atscc2-5* diplotene chromosomes appears normal, but entangled chromosomes or multivalents are observed at diakinesis. At metaphase I, *Atscc2-5* chromosomes are associated in a multivalent mass while WT meiocytes have five bivalents aligned on the equatorial plate. At anaphase, the WT homologs segregate to opposite poles, while the *Atscc2-5* meiocytes display improper chromosome segregation and chromosome fragmentation, which is consistent with previous observations in *AtSCC2-RNAi* plants [36]. In WT, the segregation of sister chromatids during meiosis II results in the formation of four nuclei. *Atscc2-5*, by comparison, produces polyads containing several micronuclei.

To further examine the *Atscc2-5* chromosome segregation defect we used fluorescence *in situ* hybridization (FISH) with a 180 bp centromeric repeat probe. We did not observe any obvious difference in the number of centromere signals between WT and *Atscc2-5* at leptotene (Fig 2B). This result confirms that duplicated sister chromatids are associated with each other at centromeric regions in mutant and WT meiocytes, which suggests that centromeric cohesin loading is initially sufficient. At pachytene, synapsis of WT homologs creates five pairs of centromere signals, while *Atscc2-5* meiocytes have more than five signals, indicating a defect in homolog paring at centromeres. Similarly, at diakinesis WT has five pairs of signals, whereas most signals in *Atscc2-5* meiocytes are unpaired. At metaphase I and anaphase I, WT centromere signals segregate to opposite poles, while *Atscc2-5* meiocytes have more than 10 centromere signals (16 shown in Fig 2B), including some that appear to lag, indicating precocious sister chromatid separation (PSCS) [39]. At metaphase II, WT has 10 paired centromere signals aligned on the equatorial plate, but *Atscc2-5* has a mixture of poorly organized, paired and unpaired signals which is also consistent with PSCS. These results suggest that the maintenance of centromere cohesin is compromised beginning in pachytene and continues through the end of meiosis.

We also analyzed chromosome morphology and dynamics of the three compound heterozygous mutant plants, and found their meiotic phenotypes are similar to the *Atscc2-5* single mutant (S6 Fig). These results demonstrate that the meiotic defects in *Atscc2-5* plants are due to a reduction of full-length AtSCC2, rather than the expression of a truncated protein.

### *AtSCC2* is required for loading meiosis-specific cohesin and genetically acts in the same pathway as *AtSYN1* and *AtWAPL1/2*

**Fig 3:**
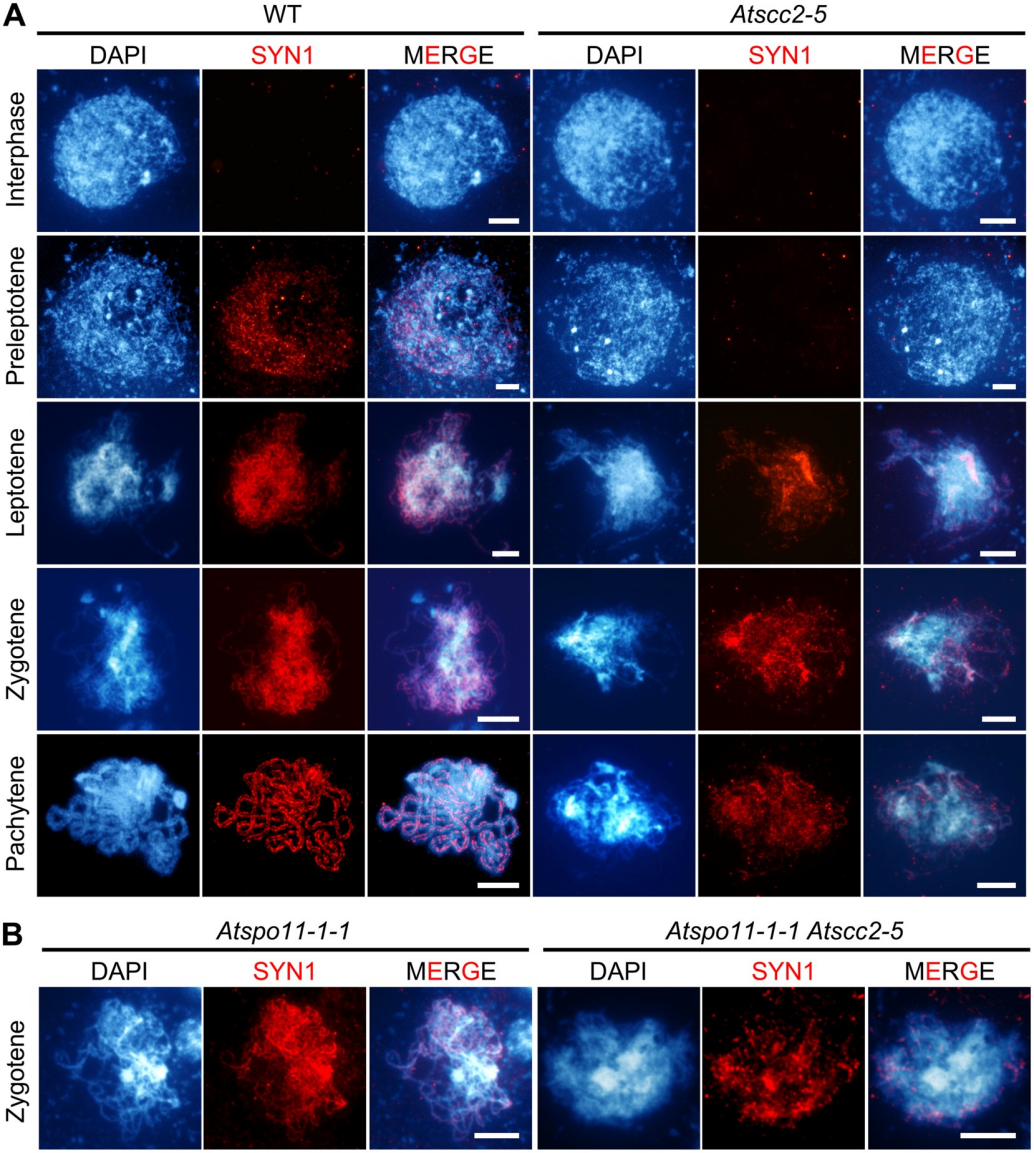
*AtSCC2* is required to load meiosis-specific cohesin subunit AtSYN1. (A) The distribution of AtSYN1 from interphase to pachytene in WT and *Atscc2-5*. Bar = 5 μm. (B) The distribution of AtSYN1 in *Atspo11-1-1* and *Atspo11-1-1 Atscc2-5* zygotene chromosomes. Bar = 5 μm.

Because SCC2 is widely reported to be responsible for loading cohesin [5], including in *Arabidopsis* [36], we used immunofluorescence staining of AtSYN1 to investigate the localization of meiosis-specific cohesin. In WT, AtSYN1 signals appear in preleptotene as diffuse foci, and extend the length of the chromosomes at leptotene (Fig 3A). Beginning at diplotene, AtWAPL1 and AtWAPL2 disassociate cohesins from chromosome arms [19, 20]. However, in *Atscc2-5*, AtSYN1 signals are barely observable at preleptotene and start to be discontinuous from leptotene onward (Fig 3A), suggesting a possible defect in the initial establishment of cohesion at centromeres and chromosome arms. It was previously reported that AtSYN1 signals in *Atspo11-1-1* and *Atscc3-1* single mutant meiocytes are similar to wild type, while AtSYN1 signals are disrupted in *Atspo11-1-1 Atscc3-1* double mutant, suggesting that the association of AtSYN1 with chromosomes needs both AtSCC3 and AtSPO11-1 [9]. Unlike the enhanced AtSYN1 defects in *Atspo11-1-1 Atscc3-1* [9], *Atspo11-1-1 Atscc2-5* double mutant exhibits similar AtSYN1 defects compared to *Atscc2-5* at zygotene (Fig 3B), indicating that *AtSPO11-1* does not play a synergetic role with *AtSCC2* in the process of AtSYN1 localization, which is consistent with the previous findings that loading of SYN1/REC8 is SPO11-1 independent in rice [40].

Since AtSYN1 is a meiosis-specific cohesin subunit and AtWAPL1/AtWAPL2 are responsible for cohesin removal in late prophase, particularly on chromosome arms, we analyzed the *Atsyn1 Atscc2-5* double and *Atwapl1-1 Atwapl2 Atscc2-5* triple mutants. The chromosome phenotypes of the *Atsyn1 Atscc2-5* double mutant (S7D Fig) are similar to *Atscc2-5* and *Atsyn1* single mutant at all stages examined (S7B and S7C Fig), including diffuse chromosomes, aberrant pachytene morphology, entangled multivalents, PSCS, and chromosome fragmentation (S7A Fig). Because AtWAPL1 and AtWAPL2 function to remove cohesin and AtSCC2 is thought to function in loading cohesin, we hypothesized that AtWAPL1/AtWAPL2 mutations might partially rescue the *Atscc2-5* meiotic defect. Unexpectedly, the *Atwapl1 Atwapl2 Atscc2-5* triple mutant is sterile and has atypical pachytene chromosomes, chromosome entanglements and chromosome fragmentation (S7F Fig), similar to the *Atscc2-5* single mutant (S7B Fig), implying that *AtSCC2*, *AtWAPL1* and *AtWAPL2* are epistatic to one another. It is possible that the PSCS observed in *Atscc2-5* is due to insufficient AtSYN1-mediated centromere cohesion in addition to the defects in chromosome arm cohesion.

### Formation of DSBs appears normal, but their repair is affected in *Atscc2-5*

**Fig 4:**
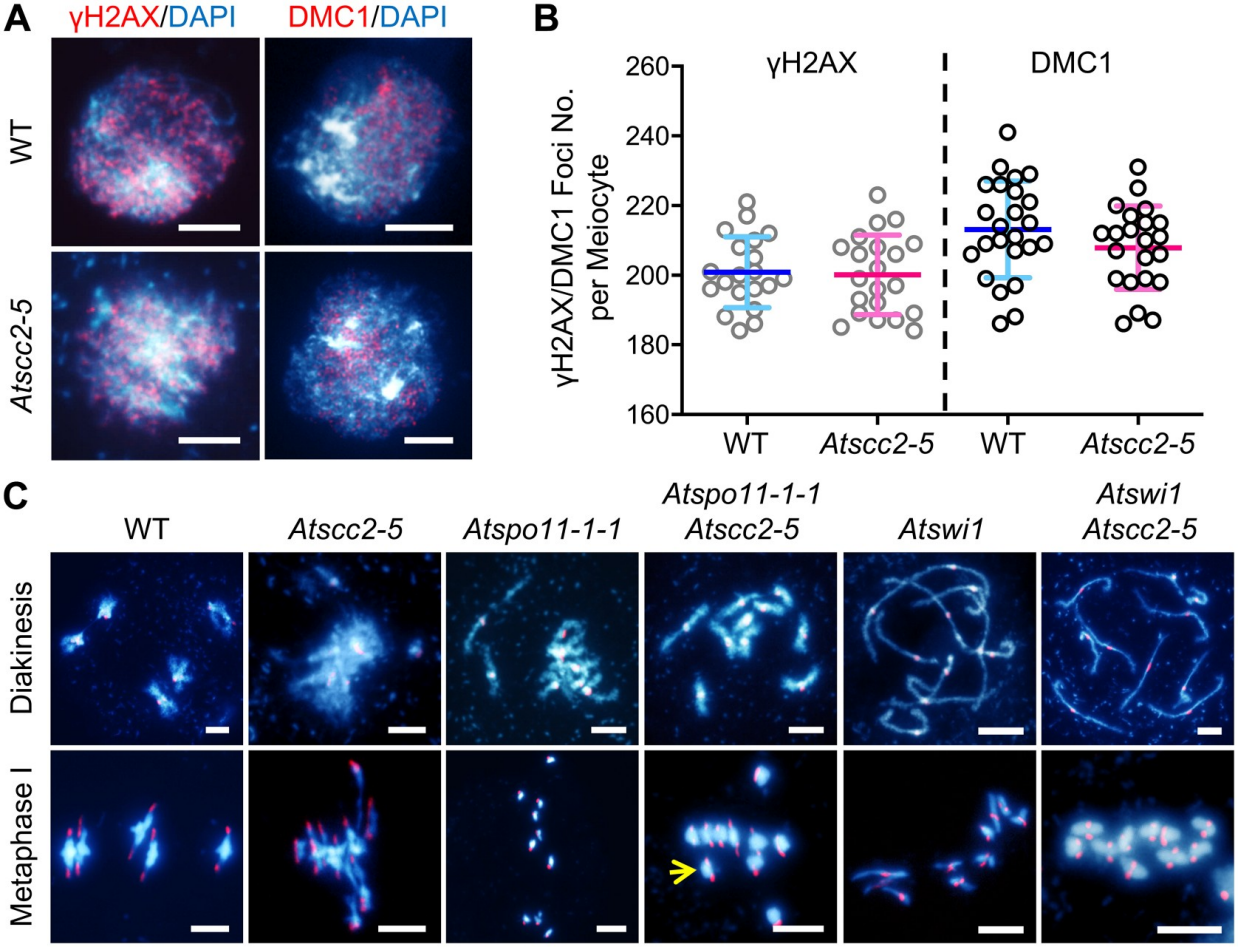
*AtSCC2* is dispensable for DSB formation but is indispensable for AtSPO11-1-dependent DSB repair. (A) Localization of γH2AX and DMC1 in WT and *Atscc2-5* zygotene male meiocytes. Bar = 5 μm. (B) Plots of the γH2AX and DMC1 foci numbers in WT and *Atscc2-5* zygotene male meiocytes (two-tailed Student’s *t* test). (C) Fluorescence *in situ* hybridization of WT, *Atscc2-5*, *Atspo11-1-1*, *Atspo11-1-1 Atscc2-5*, *Atswi1*, and *Atswi1 Atscc2-5* double mutant chromosomes using centromere probes. Yellow arrows indicate the separated centromeres of sister-chromatids at metaphase I. Bar = 5 μm.

The observation of chromosome fragmentation in *Atscc2-5* suggests that *AtSCC2* may participate in meiotic DSB repair. We examined whether the lack of AtSCC2 or chromosome-bound cohesin impacts the formation of meiotic DSBs, by using immunofluorescence staining of two DSB markers, γH2AX, a phosphorylated variant histone [41], and AtDMC1, a recombinase [42], in WT and *Atscc2-5* zygotene meiocytes (Fig 4A). We did not observe any significant difference (both p-value > 0.05, two tailed Student’s *t* test) in the number of AtDMC1 or γH2AX foci between WT (n = 21 cells for AtDMC1; n = 22 cells for γH2AX) and *Atscc2-5* (n = 24 for AtDMC1; n = 22 cells for γH2AX; Fig 4B), suggesting that *AtSCC2* is not required for DSB formation. This is consistent with the previous report in *Caenorhabditis elegans* [43].

To test whether the DSB repair defects are SPO11-1-dependent, we introduced the *Atspo11-1-1* mutation [44] into the *Atscc2-5* mutant background. *AtSPO11-1* is required for generating meiotic DSBs, and the *Atspo11-1-1* mutant has univalents at diakinesis which segregate randomly at metaphase/anaphase I (Fig 4C). The *Atspo11-1-1 Atscc2-5* double mutant has 10 unfragmented univalents at diakinesis and no multivalents at metaphase I, suggesting that *AtSCC2* participates in *AtSPO11-1*-dependent DSB repair. In addition, the metaphase I *Atspo11-1-1 Atscc2-5* univalents exhibit PSCS, possibly due to the defective AtSYN1 localization in *Atscc2-5*, providing additional evidence that centromeric cohesin between sister chromatids is compromised. *AtSWI1* is required for the establishment of sister chromatid cohesin and the initiation of meiotic recombination [18, 45]. *Atswi1* mutants have univalents which segregate randomly at metaphase I and have noticeable PSCS. In the *Atswi1 Atscc2-5* double mutants, sister chromatids are mono-oriented and there is no chromosome fragmentation, which resembles the meiotic defects in *Atspo11-1-1 Atscc2-5* double mutants. These results provide additional evidence that *AtSCC2* participates in meiotic recombination likely through loading cohesin.

**Fig 5:**
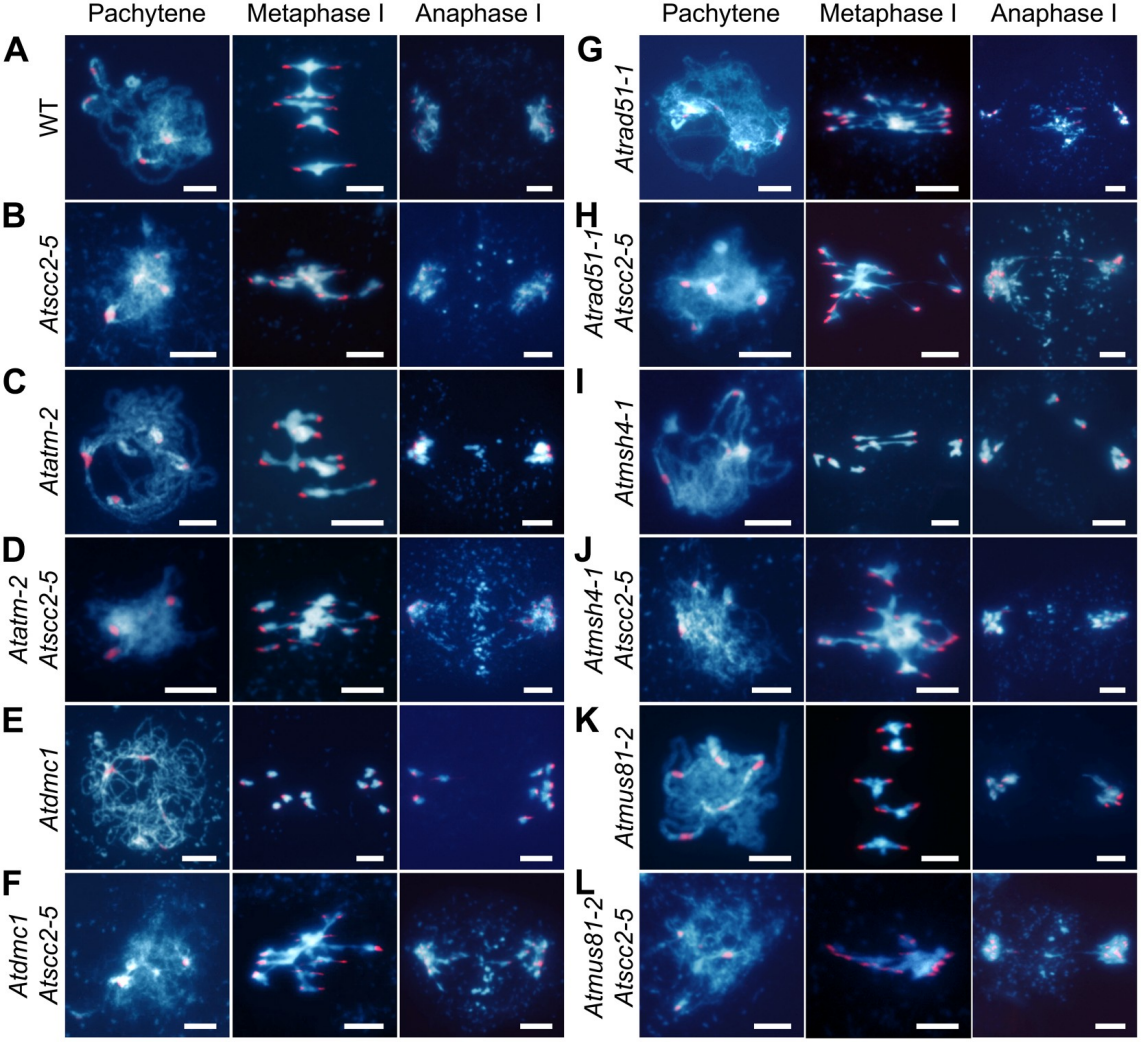
Genetic analyses of *AtSCC2* in meiotic recombination mutants. DAPI stained chromosome spreads at pachytene, metaphase I and anaphase I in (A) WT, (B) *Atscc2-5*, (C) *Atatm-2*, (D) *Atatm-2 Atscc2-5*, (E) *Atdmc1*, (F) *Atdmc1 Atscc2-5*, (G) *Atrad51-1*, (H) *Atrad51-1 Atscc2-5*, (I) *Atmsh4-1*, (J) *Atmsh4-1 Atscc2-5*, (K) *Atmus81-2*, (L) *Atmus81-2 Atscc2-5*. Bar = 5 μm.

To investigate whether *AtSCC2* has a role during other stages of meiotic recombination, we generated double mutants of *Atscc2-5* with *Atatm-2* (DSB response), *Atdmc1* (strand invasion), *Atrad51-1* (strand invasion), *Atmsh4-1* (CO resolution), and *Atmus81-2* (CO resolution) (Fig 5). Compared with *Atscc2-5* and *Atatm-2* single mutants, chromosome fragmentation is aggravated in *Atatm-2 Atscc2-5* at anaphase I (Fig 5D), suggesting that *AtSCC2* acts synergistically with *AtATM* in mediating meiotic recombination. *Atdmc1* meiocytes have 10 univalents, but no chromosome fragmentation or entanglements, presumably because AtRAD51 is able to repair DSBs using sister chromatids as a template [42]. The *Atdmc1 Atscc2-5* double mutant has chromosome entanglements at metaphase I and chromosome fragments at anaphase I (Fig 5F), similar to the *Atscc2-5* single mutant (Fig 5B), indicating that *AtSCC2* and *AtDMC1* are epistatic to one another during meiotic recombination. The *Atrad51-1 Atscc2-5* double mutant has similar severe chromosome entanglement and fragmentation phenotypes (Fig 5H) compared to the *Atrad51-1* single mutant (Fig 5G), indicating that *AtRAD51* and *AtSCC2* are epistatic to one another. Taken together, these results support the idea that AtSCC2 is required for efficient DSB repair and acts together with AtRAD51 in a manner that is distinct from the action of the meiosis-specific recombinase AtDMC1. Alternatively, because *Atscc2-5* still expresses very low levels of wild type *AtSCC2* transcript, these results could indicate that AtRAD51 is more sensitive than AtDMC1 to AtSCC2 levels.

*Arabidopsis* has two classes of CO: Type I COs are sensitive to a regulatory phenomenon called CO interference and are associated with the ZMM class of proteins, including AtMSH4; and Type II COs are insensitive to interference and are AtMUS81-dependent [46, 47]. To investigate whether *AtSCC2* is involved with one or the other, or both pathways, we compared *Atmsh4-1 Atscc2-5* and *Atmus81-2 Atscc2-5* double mutants with the corresponding single mutants. Neither *Atmsh4-1* nor *Atmus81-2* has chromosome entanglement or fragmentation phenotypes (Fig 5I and 5K). In contrast, the *Atmsh4-1 Atscc2-5* and *Atmus81-2 Atscc2-5* double mutants have similar chromosome entanglement and fragmentation phenotypes compared to *Atscc2-5* (Fig 5J and 5L), implying that *AtSCC2* likely functions upstream of *AtMSH4* and *AtMUS81*. Taken together, these data suggest that during meiotic recombination, AtSCC2 is not required for DSB formation, but is critical during steps prior to CO resolution in a manner that impinges on AtRAD51 to a greater extent than AtDMC1.

### AtSCC2 is required for axial element and synaptonemal complex (SC) formation

As described above, *Atscc2-5* zygotene chromosomes appear less condensed compared to WT (Fig 2A). It has been reported that reduced expression of *AtSCC2* affects axial element formation [36]. We used immunofluorescence staining of AtASY1, which plays important roles in the coordination of axis/SC morphogenesis [48], and AtZYP1, a component of the SC transverse elements [49] to examine axial element formation in *Atscc2-5*. Punctate AtASY1 signals are associated with chromosomes at leptotene, and then appear to be linear on chromosomes at zygotene in WT (S8A Fig). From pachytene to diakinesis, as homologous chromosomes condense, synapsis and desynapse, AtASY1 signals gradually diminish and only remain prominent in highly condensed heterochromatic regions. In *Atscc2-5* leptotene meiocytes, punctate AtASY1 signals are similar to WT, but are less concentrated, suggesting that the initial assembly of the axis is not severely compromised in *Atscc2-5*. At zygotene, AtASY1 appears as a mixture of discontinuous and linear signals, due to lack of homolog alignment and synapsis, their removal at pachytene is delayed, suggesting a defect in axis assembly completion in *Atscc2-5*. By diakinesis, AtASY1 signals in *Atscc2-5* are weaker relative to WT. These observations indicate that axis formation can initiate in *Atscc2-5*, but proceeds inefficiently and is aberrantly disassembled which further supports a defect in synapsis. The AtASY1 localization at zygotene in the *Atspo11-1 Atscc2-5* double mutant shows additive defects relative to that of the *Atscc2-5* single mutant (S8B Fig), suggesting that *AtSCC2* has a synergistic role with *AtSPO11-1* in assembly of ASY1 during meiosis, consistent with recent results reported in maize [50].

Because *Atscc2-5* plants have aberrant pachytene chromosomes and AtASY1 assembly, we speculated that their SC transverse elements may be also defective. The AtZYP1 signals in pachytene meiocytes are greatly diminished in *Atscc2-5*, compared to the linear AtZYP1 distribution on WT chromosomes (S8C Fig). Taken together, our results provide strong evidence that AtSCC2 is required for axial element assembly and SC formation.

### AtSCC2 interacts with AtSCC4 *in vivo*, but AtSCC4 is dispensable for male meiosis

**Fig 6:**
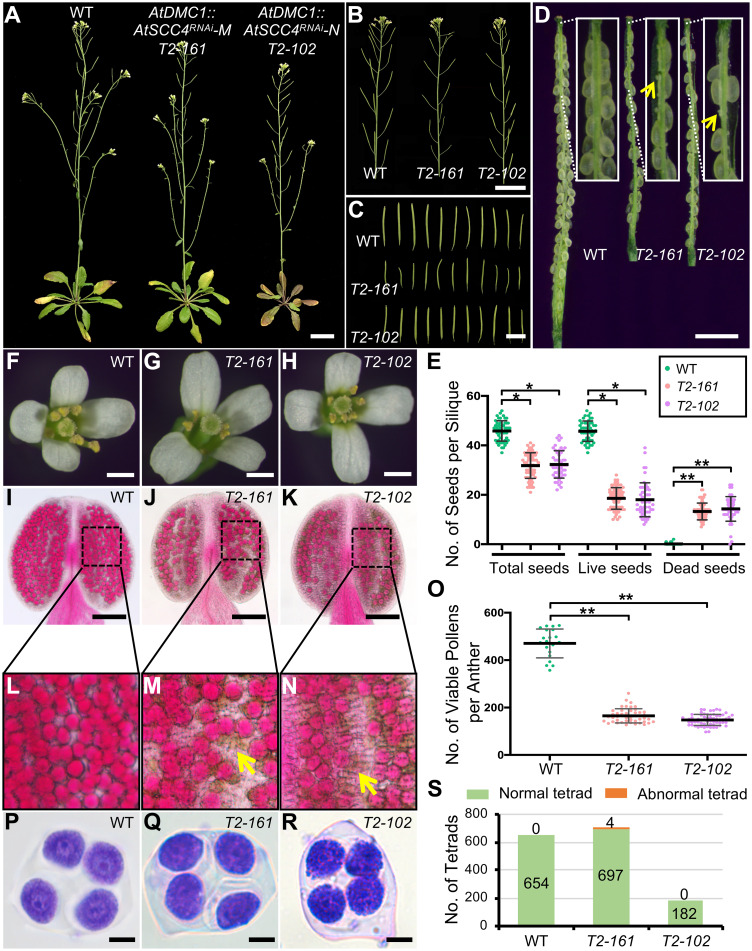
*AtDMC1*::*AtSCC4*^*RNAi*^ transgenic plants have reduced fertility. (A) WT, *AtDMC1*::*AtSCC4*^*RNAi*^*-M T2-161* and *AtDMC1*::*AtSCC4*^*RNAi*^*-N T2-102* (M and N refer the fragments used for the construction of RNAi plasmids) plants. Bar = 3 cm. (B) Primary stems of WT, *T2-161* and *T2-102*. Bar = 3 cm. (C) Siliques of WT, *T2-161* and *T2-102*. Bar = 1 cm. (D) Stripped siliques of WT, *T2-161* and *T2-102*. Yellow arrows indicate undeveloped embryos. Bar = 1 mm. (E) Plots of total seeds, live seeds and dead seeds in WT, *T2-161* and *T2-102* (* P < 0.05 or ** P < 0.01, the significance of reduced seed number in *AtSCC4*^*RNAi*^ transgenic plants versus WT, by two-tailed Student’s *t* test; each dot represents the number of seeds in one silique). (F-H) Open flowers of WT, *T2-161* and *T2-102*. Bar = 1 mm. (I-K) Alexander staining of WT, *T2-161* and *T2-102* anthers. Bar = 100 μm. (L-N) Zoom-in of WT, *T2-161* and *T2-102* pollens. (O) Plots of viable pollen in WT, *T2-161* and *T2-102* (** P < 0.01, the significance of reduced pollen number in *AtSCC4*^*RNAi*^ transgenic plants versus WT, by two-tailed Student’s *t* test; each dot represents the number of pollen in one anther). (P-Q) Toluidine blue dye staining of WT, *T2-161* and *T2-102* tetrad-stage microspores. Bar = 100 μm. (S) Plots of normal and abnormal tetrad-stage microspores in WT, *T2-161* and *T2-102*.

SCC2 has been reported to interact with SCC4 in several organisms, including humans, maize and *Arabidopsis* [25, 27, 35]. Our yeast two-hybrid assay also confirmed their physical interaction (S9A and S9B Fig). The interaction was further validated by bimolecular fluorescence complementation (BiFC) in tobacco cells (S9C Fig). A recent study demonstrated that the N terminus of AtSCC2 (1–824 aa) interacts with AtSCC4 [27]. To further refine the specific interacting regions, we divided the N-terminus into AtSCC2N1 (1–254 aa) and AtSCC2N2 (255–427 aa) (S9A Fig) and found that only AtSCC2N1 interacts with AtSCC4 (S9B Fig). Because *AtSCC4* is essential in mitosis [27], we used a tissue-specific RNAi strategy to test its role in meiosis by targeting two *AtSCC4* regions expressed from the meiosis-specific *AtDMC1* promoter (*AtDMC1*::*AtSCC4*^*RNAi*^*-M* and *AtDMC1*::*AtSCC4*^*RNAi*^*-N*; S9D and S9E Fig), and obtained 87 positive transformants, including 37 *AtDMC1*::*AtSCC4*^*RNAi*^*-M* and 50 *AtDMC1*::*AtSCC4*^*RNAi*^*-N* plants. Five *AtDMC1*::*AtSCC4*^*RNAi*^*-M* plants and ten *AtDMC1*::*AtSCC4*^*RNAi*^*-N* plants had relatively normal vegetative growth, but reduced fertility. We selected 8 sterile lines for subsequent study. The pollen viability and number of seeds in all 8 lines were significantly reduced compared to WT (n = 10, S10A and S10B Fig). qRT-PCR revealed that *AtSCC4* expression levels in the meiocytes of the RNAi lines are significantly reduced compared to WT (p-value < 0.01, two-tailed Student’s *t* test, S10C Fig). We further analyzed two representative RNAi lines (*T2-102* and *T2-161*), which have reduced fertility, short siliques, few seeds and less viable pollen relative to WT (Fig 6A–6O), but their tetrad-stage microspores have no obvious differences compared to WT (Fig 6P–6S). Analysis of male meiotic chromosome spreads confirmed that stages in the RNAi plants were similar to WT (S11 Fig). This result suggests that AtSCC4 does not play a prominent role in male meiosis, but it is formally possible that the residual gene product, after knocking down gene expression by 90%, is sufficient for wild type function. The RNAi plants appeared to produce sufficient pollen to allow pollination, so we hypothesized that female fertility may be impaired in *AtSCC4*^*RNAi*^ plants. To test this hypothesis, we reciprocally crossed the WT and *AtSCC4*^*RNAi*^ plants. WT pistils pollenated with *T2-161* or *T2-102* pollen produced indistinguishable seeds per silique respectively compared with WT (S3 Table and S12A Fig). As female parents the transgenic plants produced only 15.9 and 14.6 normal seeds, respectively (S3 Table and S12A Fig). However, no obvious female meiotic defects were observed in WT (n = 159), *T2-102* (n = 86) or *T2-161* (n = 54) (S12B Fig). A previous study showed that AtSCC4 is required for embryo development [27]. Consistently, 11.8% (n = 17) *T2-161* and 28.6% (n = 14) *T2-102* embryos exhibited asymmetric cell division at globular stage compared with WT (n = 15) (S12C Fig). Taken together, these results suggest that *AtSCC4* is dispensable for male and female meiosis. It is possible that the *Arabidopsis* SCC2-SCC4 complex is only required for loading cohesin during mitosis, while *AtSCC4* does not play a role in meiosis.

### The AtSCC2 PHD domain binds to histones *in vitro*

Among SCC2 homologs, only those of plants contain a plant homeodomain (PHD) with a C4-H-C3 amino acid motif (S13A and S13B Fig). Phylogenetic analysis demonstrates that the SCC2 PHD domain is highly conserved in land plants, but absent in the green algae *Chlamydomonas reinhardtii*, *Volvox carteri* and *Micromonas* (S13B Fig), suggesting that the SCC2 PHD domain may have played an important role during the adaptation of plants to terrestrial environments.

**Fig 7:**
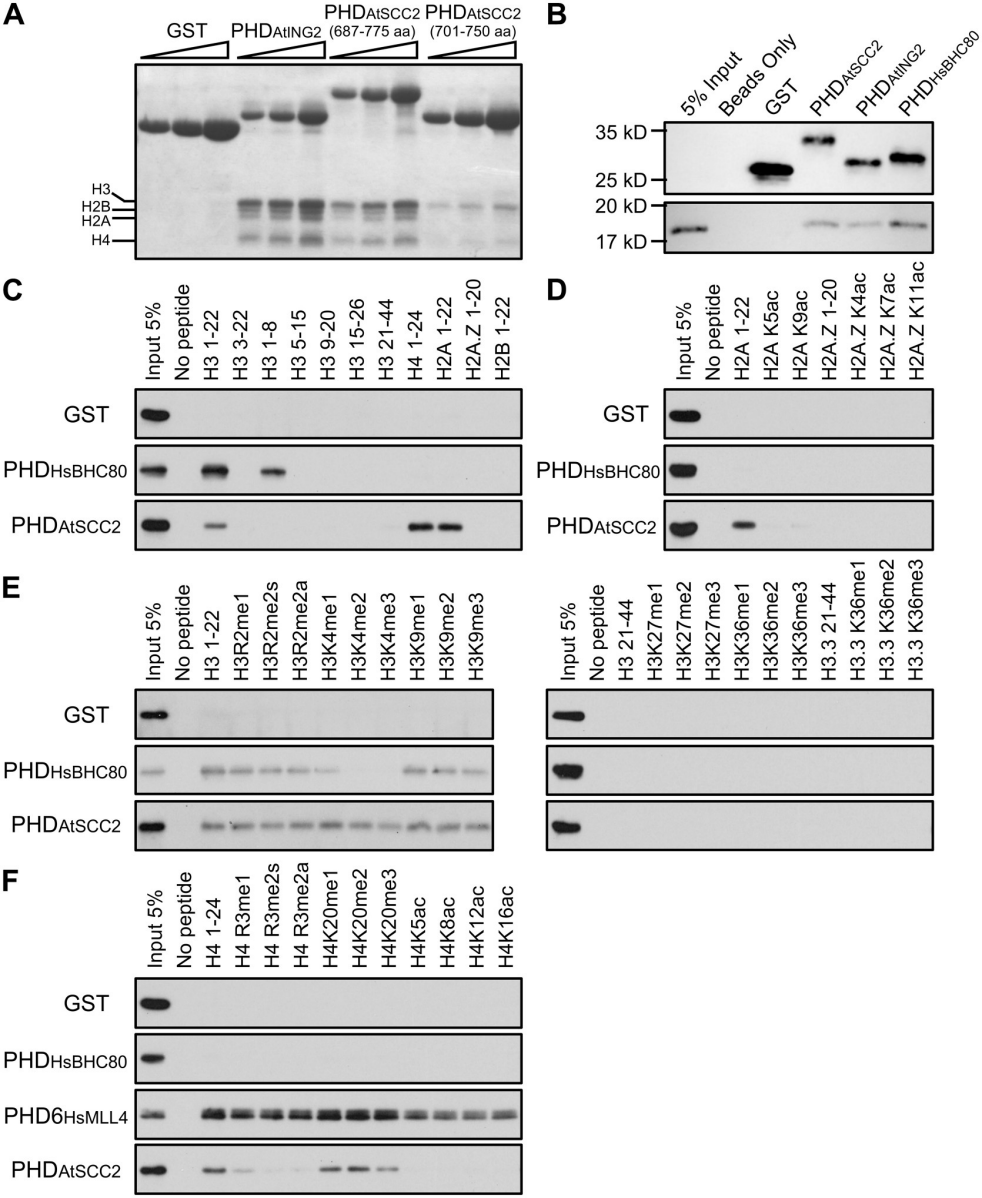
AtSCC2 PHD can bind to histones. (A) Pull-down assay of GST, GST-AtING2_PHD_, GST-AtSCC2_PHD (687–775 aa)_, GST-AtSCC2_PHD (701–750 aa)_ with calf thymus histones. (B) Pull-down assay of GST, GST-HsBHC80_PHD_, GST-AtSCC2_PHD (687–775 aa)_ with H3. (C) Pull-down assay of GST, GST-HsBHC80_PHD_, GST-AtSCC2_PHD (687–775 aa)_ with different histone peptides in N terminal length. (D) Pull-down assay of GST, GST-HsBHC80_PHD_, GST-AtSCC2_PHD (687–775 aa)_ with unmodified and modified H2A peptides. (E) Pull-down assay of GST, GST-HsBHC80_PHD_, GST-AtSCC2_PHD (687–775 aa)_ with unmodified and modified H3 peptides. (F) Pull-down assay of GST, GST-HsBHC80_PHD_, GST-AtSCC2_PHD (687–775 aa)_ with unmodified and modified H4 peptides.

Some PHD domains can bind to unmodified H3K4 or methylated H3K4 in animals and plants [51, 52]. To investigate the potential histone binding specificity of the AtSCC2 PHD domain, we aligned several plant SCC2 PHD sequences and compared them to PHD domains with known histone binding targets (S13C Fig). PHD domains that recognize methylated H3K4 possess three aromatic amino acids (Y-Y-W), but these are absent in the AtSCC2 PHD domain, suggesting that AtSCC2 may not bind methylated H3K4. Based on the alignments, the AtSCC2 PHD domain is more similar to the human BHC80 PHD domain which can bind unmodified H3K4 [53]. To test whether the AtSCC2-PHD has a similar binding affinity, we used an *in vitro* pull-down assay of two different length AtSCC2-PHD constructs (701–750 aa and 687–775 aa) and a known AtING2 PHD domain as positive control [52] with calf thymus histones. The result showed that the AtSCC2-PHD is able to bind to histones, similar to the AtING2-PHD positive control, but the longer AtSCC2-PHD has a stronger binding affinity than the shorter one (Fig 7A). The pull-down assay using H3 from calf thymus showed AtSCC2-PHD can bind to H3 (Fig 7B). We further tested AtSCC2-PHD with different length unmodified histone peptides. As expected, the HsBHC80-PHD positive control was able to bind to N terminal unmethylated H3 peptides. In contrast, AtSCC2-PHD was able to bind either H3 1–22, H2A 1–22 or H4 1–24 peptides, but not H2A variant H2A.Z (Fig 7C). Binding assays with modified histone H3, H4 and H2A tails showed that methylation does not affect the binding affinity, but acetylation inhibits binding (Fig 7D–7F). These results suggest that the AtSCC2 PHD domain may recognize intact histone octamers.

### The AtSCC2 PHD domain is required for meiosis but not mitosis *in vivo*

**Fig 8:**
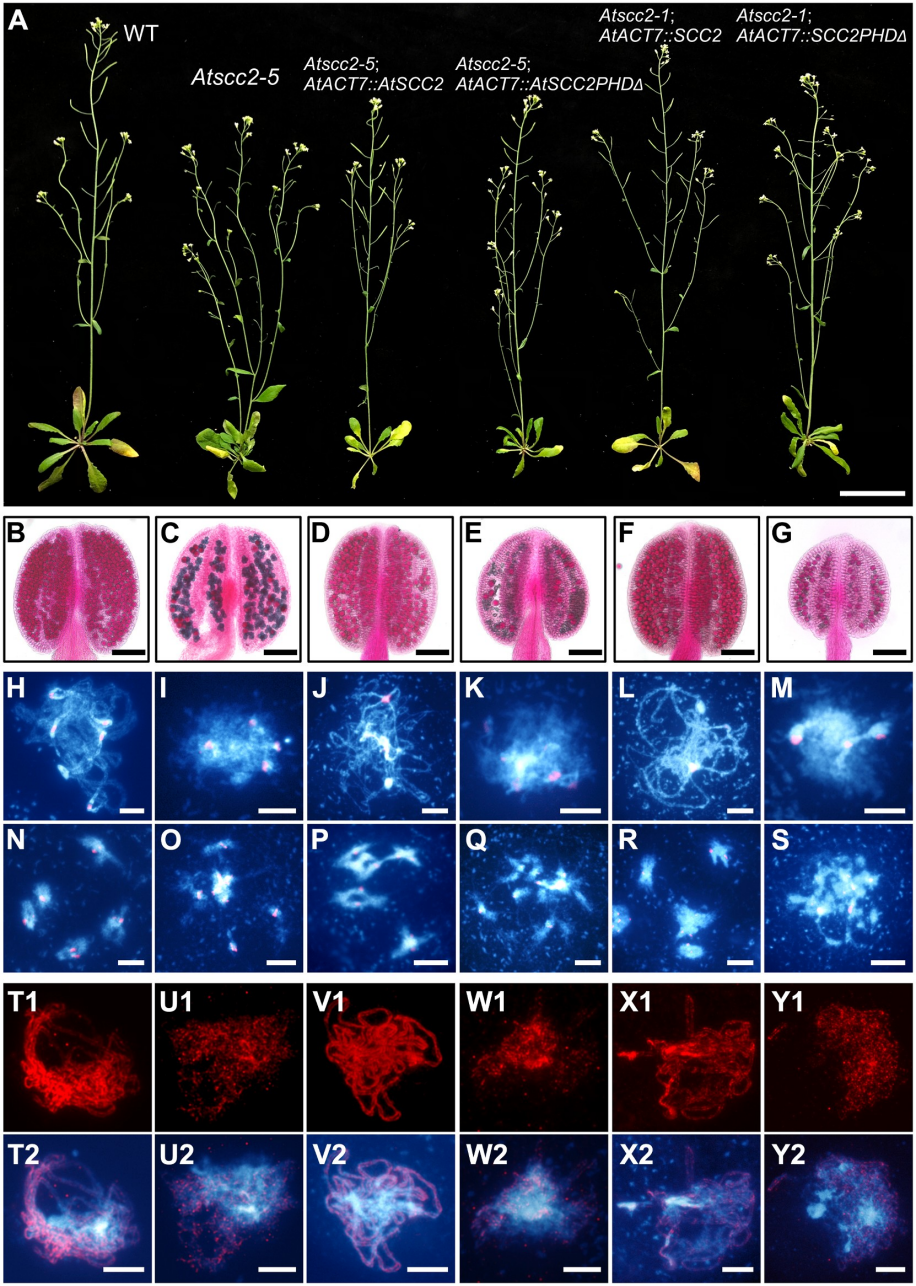
The AtSCC2 PHD domain is indispensable for male meiosis but not mitosis. (A) WT, *Atscc2-5*, *Atscc2-5*; *AtACT7*::*AtSCC2*, *Atscc2-5*; *AtACT7*::*AtSCC2PHDΔ*, *Atscc2-1*; *AtACT7*::*AtSCC2* and *Atscc2-1*; *AtACT7*::*AtSCC2PHDΔ* plants. Bar = 3 cm. (B-G) Alexander staining of the corresponding anthers. Bar = 100 μm. (H-S) DAPI stained chromosome spreads and FISH with centromere probes at pachytene and diakinesis from the plants shown in panel A. Bar = 5 μm. (T1-Y2) Distribution of AtSYN1 signal at pachytene in meiocytes from the corresponding plants. Bar = 5 μm.

To test the function of the AtSCC2 PHD domain *in vivo*, we transformed *Atscc2-5*^*+/-*^ (*Atscc2-5*^*-/-*^ is a weak allele) and *Atscc2-1*^*+/-*^ (*Atscc2-1*^*-/-*^ is a null allele) mutant plants with constructs encoding full-length AtSCC2 and AtSCC2-PHDΔ (PHD domain deletion in 702–745 aa) expressed from the ubiquitous *AtACT7* promoter (Fig 8). qRT-PCR confirmed that the *AtACT7*::*AtSCC2* and *AtACT7*::*AtSCC2PHDΔ* transgenes are expressed in four representative lines, compared to WT and *Atscc2-5* mutant controls (S14 Fig). Neither AtSCC2 nor AtSCC2-PHDΔ in *Atscc2-5* mutant background (called *Atscc2-5*; *AtACT7*::*AtSCC2* and *Atscc2-5*; *AtACT7*::*AtSCC2PHDΔ*) affect vegetative growth of the transgenic plants compared to WT or *Atscc2-5* controls (Fig 8A). However, *AtACT7*::*AtSCC2* is able to rescue (Fig 8D, 8J, 8P and 8V1) the fertility and aberrant meiotic phenotypes of *Atscc2-5* (Fig 8C, 8I, 8O and 8U1). In contrast, *AtACT7*::*AtSCC2PHDΔ* is not able to rescue the meiotic phenotypes (Fig 8E, 8K, 8Q and 8W1), suggesting that the PHD domain is required for the meiotic functions of *AtSCC2*.

Because the *Atscc2-1* null allele is embryonic lethal, we examined whether the AtSCC2 PHD domain is also essential for mitosis, and found that *Atscc2-1*; *AtACT7*::*AtSCC2* transgenic plants have normal vegetative growth, fertility and meiotic phenotypes (Fig 8A, 8F, 8L, 8R and 8X1), similar to WT. In contrast, *Atscc2-1*; *AtACT7*::*AtSCC2PHDΔ* transgenic plants also have normal vegetative growth, but have reduced fertility, increased pollen inviability, and aberrant meiotic phenotypes (Fig 8G, 8M, 8S and 8Y1), similar to *Atscc2-5*. These results provide *in vivo* evidence that the AtSCC2 PHD domain is required for meiosis and fertility, but not for vegetative development.

To further develop a mechanistic understanding of the AtSCC2-PHD domain in meiosis, we modeled the structure of full length AtSCC2 using the SWISS-MODEL web server (https://www.swissmodel.expasy.org) [54]. The crystal structure of *Chaetomium thermophilum* SCC2 (PDB code: 5T8V) was chosen as the template with the highest rank to build the predicted model. *C*. *thermophilum* and *Arabidopsis* SCC2 share 19.5% sequence identity. The results showed that AtSCC2 full length protein can fold into a hook-like structure (S15A Fig) that is thought to be important for cohesin loading function both *in vitro* and *in vivo* [26, 55, 56]. The structure also presents the PHD domain on the surface (S15A Fig), providing the possibility that it is available to bind to histones. We also modeled the uncorrected spliced transcript of *Atscc2-5*, which produces a protein with a severely attenuated hook-like structure which we speculate is incapable of mediating cohesin loading (S15B Fig).

## Discussion

Cohesin is a large protein complex that participates in multiple biological processes including DNA replication, DNA repair, chromosome segregation and gene expression [5]. The cohesin loader SCC2-SCC4 complex has been well studied in yeast [5]. However, because SCC2 is essential in multicellular organisms, including plants [36], its function in both mitosis and meiosis remains unclear. Previous analyses that knocked down *AtSCC2* expression revealed some meiotic functions [36], but the underlying molecular mechanism is still unclear. In this study, we generated a hypomorphic allele of *AtSCC2* to comprehensively analyze the role of AtSCC2 in meiosis. We found that *AtSCC2* is required for AtSPO11-1- and AtRAD51-dependent meiotic DNA repair and works synergistically with AtATM. We also showed that meiotic AtSCC2-mediated cohesin loading may not require AtSCC4, and that AtSCC4 is not required for meiosis. Finally, we showed that *AtSCC2* is indispensable for loading AtSYN1 during meiosis, likely via its PHD domain, and that the PHD domain is required for meiosis but not mitosis.

### A proposed model for AtSCC2 meiotic function

Cohesin loading requires the SCC2-SCC4 complex in several organisms including plants. Previous studies showed that AtSCC4 interacts with AtSCC2, and its localization on chromosomes is AtSCC2-independent [27], probably depending instead on chromosome remodeling proteins [35]. Furthermore, AtSCC4 is required for loading the mitotic cohesin subunit AtSYN4 into chromosomes [27]. Based on previous findings and our data, we proposed a model to show how AtSCC2 functions in meiosis (S15C Fig). In wild type, at preleptotene, meiosis-specific cohesin is gradually loaded onto chromosomes, which requires AtSCC2. Because AtSCC4 is dispensable for meiosis, we hypothesize that AtSCC2 uses its PHD domain binding histones to determine the SYN1 loading sites. After leptotene, meiosis-specific cohesin is fully localized on chromosomes. In the *Atscc2-5* mutant, the truncated AtSCC2 retains its N-terminal PHD domain but lacks the C-terminal hook (S15B Fig) rendering it incapable of cohesin loading. *Atscc2-5* still produces a residual amount of full-length protein which is efficient for mitotic cell divisions, perhaps due to the action of AtSCC4. This is similar to the findings in yeast where a 70% reduction in mitotic cohesin levels impedes DNA repair but is still sufficient to support chromosome segregation [57]. However, in *Atscc2-5*, at similar stages, the presence of only residual AtSCC2 significantly reduces the efficiency for AtSYN1 loading (S15C Fig), leading to multiple meiotic defects.

### The importance of the SCC2 PHD domain in land plant

PHD fingers are structurally conserved modules found in a variety of proteins including those that modulate gene expression [58]. They are comprised of 50–80 amino acids that typically form a two-stranded, anti-parallel β-sheet, a C terminal α-helix and a Cys4–His–Cys3 motif which coordinates two zinc cations [51]. Many PHD fingers are able to recognize the N terminal tail of histone H3, including unmethylated and methylated H3K4, H3R2 and acetylated H3K14 [51]. In *Arabidopsis*, only two PHD finger containing proteins, AtMMD1/AtDUET and AtSCC2, are known to be required for meiosis [36, 59, 60]. The PHD finger of AtMMD1/AtDUET is unique and highly conserved in plants and can bind to methylated H3K4, thereby regulating *AtTDM1* and *AtCAP-D3* gene expression [59, 60]. As described above, the SCC2 PHD finger is also conserved in land plants, but not in algae, animals or fungi (S13 Fig). Land plants evolved from an ancestral charophycean alga about 450 million years ago and dominate the terrestrial environment [61]. We speculate that SCC2 may have acquired its PHD finger during the evolutionary transition of plants from aquatic to terrestrial environments. Unlike *Arabidopsis* and *Oryza sativa*, our understanding of algal meiosis is still limited [62], making difficult to interpret the conservation and divergence of PHD domain between algae and higher plants. One possibility is that, algal meiosis is mechanistically more similar to the processes in ancestral species in which meiosis first evolved and may not need the replacement of mitosis-specific cohesin by a meiosis-specific one [63]. We also cannot exclude the possibility that SCC2-PHD domain functions diverge among plant taxa. It is notable that SCC2 has acquired other lineage-specific domains over evolutionary time, especially on its N terminus. Recently, human SCC2 was found to participate in some biological process via its N terminal, independently of SCC4. Human HP1 recruits SCC2 by interacting with its N terminus (996–1009 aa, HP1-interacting motif) in a SCC4-independent manner during DNA damage repair [64], while this motif is not existed in *Arabidopsis* SCC2.

In our study, we found the AtSCC2 PHD domain, independently of AtSCC4, directly binds to calf histones and N terminal region of H2A, H3 and H4 (Fig 7). Furthermore, *in vivo* evidence supports the idea that the AtSCC2 PHD domain is important for fertility, meiosis and cohesin loading. The mechanisms we identified, involving the SCC2 PHD domain in meiosis and/or cohesin loading, are likely conserved at least in plants.

### Conservation and divergence of SCC2 in cohesin loading across species

*Arabidopsis* SCC2 is required for loading the cohesin subunits AtSYN1 (in our study) and AtSCC3 onto meiotic chromosomes [36]. In yeast and animal mitosis, SCC2 always works together with SCC4 and SCC4 seems to determine the location of cohesin binding along chromosomes. It has been reported that the recruitment of SCC2-SCC4 onto chromosomes may depend on either the pre-replication complex during S phase or chromosome remodeling complex [32, 34, 35, 65, 66]. In *Xenopus*, the recruitment of SCC2 onto chromosomes depends on MCM2-7 [32, 65]. Similar mechanisms may also exist in humans [34]. In budding yeast, RSC (remodels the structure of chromatin) can facilitate the loading of cohesin onto chromosome arms [66]. Recently, maize SCC4/Dek15 was found to be able to interact with several chromosome remodeling proteins, providing additional potential for SCC4-dependent SCC2 recruitment [35]. Compared to mitosis, our understanding of meiotic SCC2 recruitment is much less complete. Here, we provide several lines of evidence that *Arabidopsis* SCC2 has a unique PHD domain that is required for meiosis, while AtSCC4 is dispensable for meiosis, supporting a distinct role of SCC2-SCC4 in plant meiosis compared with other organisms.

## Materials and methods

### Plant material and genotyping

The *Atscc2-5* mutant was isolated from an EMS-mutagenized mutant library of FTL interval “I3” (CFP and DsRed transgenes insertions on chromosome 3 in Col-3 background) [67]. Wild type (Col-0), *Atspo11-1-1* [44], *Atscc2-1* (SALK_151009) [36], *Atscc2-3* (SALK_052585) [36], *Atscc2-4* (SALK_079431), *Atatm-2* (SALK_006953) [68], *Atdmc1*, *Atrad51-1* (GABI_134A01) [69], *Atmsh4-1* (SALK_136296) [46], *Atmsu81-2* (SALK_107515) [70], *Atwapl1-1 atwapl2* [19], *Atsyn1* (SALK_137095) [71] and *Atswi1* (SAIL_654_C06) [20] used in this study were genotyped using PCR primers as described in S4 Table.

### Growth conditions

Plants were cultivated in a growth chamber under a 16-hour day/8-hour night photoperiod, at 20°C with 70% humidity. For *in vitro* culture, seeds were sterilized with 70% ethanol and plated on 1/2 Murashige and Skoog medium (MS medium). After incubation for 48 hours at 4°C in the dark, plants were then transferred to soil and cultivated in a growth chamber.

### Mutagenesis

EMS mutagenesis was performed as described previously [72]. Briefly, 120 mg of seeds were incubated with gentle agitation at room temperature for 16 hours in 45 mL ddH_2_O with 0.27% ethylmethane sulfonate (EMS). Mutagenized seeds were rinsed twice with 45 mL water for 4 hours followed by 9 additional 45 mL rapid rinses. Rinsed seeds were suspended in 45 mL of 0.05% agarose and incubated at 4°C for 3 days. The cold treated seeds were transferred to 100 mL of fresh 0.05% agarose solution and planted on soil.

### Cloning of *AtSCC2* by whole genome sequencing

*Atscc2-5* was crossed with L*er* to acquire mapping populations. Genomic DNA was extracted from 97 sterile F2 progeny and mixed. The bulked DNA was sequenced on an Illumina HiSeq 3000 platform, providing 48 million 150-bp paired-end reads (7.2 Gb, ~ 60X coverage). We downloaded the 2X100 bp paired-end whole genome resequencing datasets of Col (SRX202246, 9.6 Gb, ~ 80X coverage) and L*er* (SRX202247, 8.4 Gb, ~ 70X coverage) from the NCBI SRA database [73]. The raw reads of Col, L*er* and the F2 bulk were trimmed to remove potential adapter and low-quality sequences using Trimmomatic 0.36 [74] with the parameter “LEADING:3 TRAILING:3 SLIDINGWINDOW:4:15 MINLEN:50”. The filtered short reads were mapped onto the TAIR10 *Arabidopsis thaliana* (Col) reference genome [75] by BWA [76]. To obtain Single-nucleotide polymorphism (SNP) markers between Col and L*er*, we collected SNPs from both the 1001 Genomes project website (http://1001genomes.org/projects/MPISchneeberger2011/index.html) and the mapping results of Col and L*er* reads by using inGAP [77]. inGAP-sv [78] was employed to detect larger-scale structural variants. The SNPs were examined using the methods described by Qi *et al*. [79] to avoid artificial variants from false mapping of non-allelic reads. To identify candidate regions that capture causal mutations, we used a sliding window analysis to estimate allelic ratios, with a window size of 100 kb and a sliding step of 50 kb. Novel SNPs that exhibit G-A or C-T nonsynonymous substitutions in the “valley” regions were considered as candidate causal mutations for subsequent analysis.

### qRT-PCR for transcript expression analysis

Total RNAs were extracted from meiocytes or core inflorescences using Trizol reagent (Invitrogen, USA). cDNA synthesis was performed using PrimeScript RT with gDNA Eraser (Takara, Japan) following the manufacturer’s instructions. qRT-PCR was performed using iTaq Universal SYBR Green supermix (Bio-Rad, USA) and the gene expression level was calculated employing the 2^-ΔΔCt^ method [80]. *AtTIP41-like* gene was chosen as the reference gene as previously reported [81]. Each qRT-PCR experiment had three biological replicates and the statistical significance (p-values) of differences in gene expression levels between samples was analyzed using a two tailed Student’s *t* test. The qRT-PCR primers used are listed in S3 Table.

### Plasmid construction and plant transformation

For complementation plasmid construction, the full-length CDS of *AtSCC2* or two separated fragment CDS of *AtSCC2* lacking PHD domain (*AtSCC2ΔPHD*) was cloned into modified plasmid pCAMBIA1306 (*AtACT7*::3*FLAG) by One-step Cloning Kit (Novoprotein, China). To generate transgenic *AtSCC4*-RNAi plants, two regions of *AtSCC4* CDS were amplified using PCR with the primers including *Nco*I/*Xba*I and *Spe*I/*Sal*I restriction sites, respectively. The amplification products were ligated into the pMeioDMC1-intron vector using *Spe*I/*Nco*I for the sense fragment and *Sal*I/*Xba*I for antisense fragment. The constructs were then individually transformed into *Agrobacterium tumefaciens* GV3101 (Weidi, China) and bacterial cultures were used for dip transformation as previously reported [82]. Positive T1 plants were screened on 1/2 MS medium containing 25 mg/L hygromycin.

### Morphological analysis of plants

Whole plants, stems and siliques were photographed using a Canon digital camera SX20 IS (Canon, Japan). Images of dissected seedpods were taken using a Zeiss Stereo Discovery microscope (Zeiss, Germany). Pollen viability was analyzed via modified Alexander red staining at 65°C for 40 min [83]. Tetrad-stage microspores were stained with toluidine blue dye as previously described [84]. Images of tetrad and mature pollen were collected using a Zeiss Axio Scope A1 microscope (Zeiss, Germany). Excel 2018 (Microsoft, USA) was used to calculate the significances (p-values) of seed numbers and pollen numbers between WT and *AtSCC4*^*RNAi*^ transgenic plants using a two tailed Student’s *t* test.

### Embryo morphogenesis observation

Siliques were fixed in Carnoy’s fixative for more than one hour at room temperature. Wash the fixed siliques three times with ddH_2_O. Seeds were taken out of the siliques, incubated on a sample glass in chloral hydrate solution (4 g chloral hydrate, 1 mL glycerol, 2 mL water) for 3–5 min and covered with a cover slip. The embryos were observed with DIC optics using the AxioScope A2 microscope.

### Cytological analysis

Chromosome spreading, fluorescence in situ hybridization (FISH), and immunofluorescence staining were all conducted following the procedures as described previously [84]. Rabbit-sourced polyclonal AtASY1 antibody and rat-sourced AtZYP1 antibody were used at a 1:200 dilution in blocking buffer as previously described [85]. The rabbit-sourced polyclonal AtDMC1 antibody was used at a 1:500 dilution as previously described [86]. The rabbit-sourced polyclonal AtSYN1 antibody was newly generated (Shanghai Ango Biotechnology CO, China) and used at a 1:200 dilution. The secondary antibodies Alexa Fluor 488 Goat Anti-Rat IgG (H+L) (A-21208) and Alexa Fluor 555 Goat Anti-Rabbit IgG (H+L) (A-21428) (Invitrogen, USA) were used at 1:500 and 1:1000-fold dilutions, respectively. All cytological images were taken using a Zeiss Axio Scope A1 microscope (Zeiss, Germany). Statistical analysis of the significance (p-values) of the differences in the number of AtDMC1 and AtγH2AX foci between WT and *Atscc2-5* was done using a two tailed Student’s *t* test.

### Western blot

In brief, total proteins were extracted using a protein extraction buffer (20 mM Tris-HCl pH 8.0, 150 mM NaCl, 1 mM EDTA, 10% glycerol, 1 mM PMSF) mixed with inflorescences ground to a fine powder in liquid nitrogen. The supernatant was for SDS-PAGE electrophoresis after 3 h incubation at 4°C and 30 min centrifugation at 12,000 rpm. Proteins were transferred to nitrocellulose (NC) membranes (Abm, China) and incubated in monoclonal anti-FLAG antibody (GNI, Japan) at a 1:1000 dilution. HRP-conjugated anti-mouse antibody (1:5000, GNI, Japan) was used as the secondary antibody. Protein–antibody conjugates were revealed using Clarity Western ECL Substrate (Bio-Rad, USA) according to the manufacturer’s protocol.

### Histone peptide binding assay

*AtSCC2* cDNA regions encoding different length PHD finger (residues 687–775 and 701–750) were cloned into the pGEX 4T-1 vector using *BamH*I/*Sal*I restriction sites. Constructs were transformed to *E*. *coli* Rosetta (DE3). GST-fusion proteins were induced by 0.02 mM/L IPTG and purified using GST•Bind Resin (Merck, Germany). Biotinylated histone peptides were synthesized at Beijing Scilight Biotechnology Ltd. Co. or purchased from Millipore. Briefly, 1 μg of peptides were incubated with 2 μg of GST-fusion protein in 300 μL binding buffer (50 mM Tris-HCl, pH 7.5, 150 mM NaCl, 0.05% (v/v) NP-40, 1 mM phenylmethylsulphonyl fluoride (PMSF)) for two hours at 4°C. 5 μL Streptavidin magnetic beads (Pierce) were added, followed by another 1 h of incubation at 4°C. After washing three times with binding buffer, the beads were boiled and subjected to SDS-PAGE and WB.

### Phylogenetic tree construction

The construction of the green plant phylogenetic analysis was mainly based on two previous studies [87, 88].

### Accesion numbers

*Arabidopsis* Genome Initiative (AGI) gene identifiers used in this study are as follows: *AtSCC2* (*AT5G15540*), *AtSCC4* (*AT5G51340*), *AtSPO11-1* (*AT3G13170*), *AtASY1* (*AT1G67370*), *AtZYP1* (*AT1G22260*), *AtRAD51* (*AT5G20850*), *AtDMC1* (*AT3G22880*), *AtSYN1* (*AT5G05490*), *AtATM* (*AT3G48190*), *AtMSH4* (*AT4G17380*), *AtMSU81* (*AT4G30870*), *AtSWI1* (*AT5G51330*), *AtWAPL1* (*AT1G11060*), *AtWAPL2* (*AT1G61030*) and *AtTIP41-like* (*AT4G34270*).

## Supporting information

S1 Fig Isolation of a male sterile mutant *line88*. (A) The rosette leaves of four-week old WT and *line88*. Bar = 3 cm. (B) Comparison of an eight-week old WT plant and a *line88* mutant plant. Bar = 3 cm. (C) Comparison of the WT and *line88* stems. The yellow arrow indicates a short silique in *line88*. Bar = 3 cm. (D) The first 18 siliques of WT and *line88*. Bar = 1 cm. (E and F) The open flowers of WT and *line88*. Bar = 1 mm. (G and H) WT and *line88* pollens stained with Alexander dye. Bar = 100 μm. (I and J) WT and *line88* tetrads stained with Toluidine blue dye. Red arrows indicate the micronuclei. Bar = 5 μm.
(TIF)

S2 Fig Identification of genomic region putatively harboring causal mutation for *line88* by using a mapping-by-sequencing strategy. (A) Genotypic ratios are evaluated on sliding windows of 100-kb with step of 50-kb. The candidate genomic region is marked by a pink bar. (B) Distribution of genotypic ratio of SNPs in 4,403,200–5,324,800 bp on chromosome 5. (C) Mapping details of resequencing reads from *line88* along with causal mutation (red triangle) on *AT5G15540* gene.
(TIF)

S3 Fig The AtSCC2 complementation rescues the sterile and meiotic defects in *Atscc2-5* background. (A) Comparison of a WT plant, *Atscc2-5* and *Atscc2-5*; *AtACT7*::*AtSCC2* transgenic plant. Bar = 3 cm. (B) Comparison of the stems of WT, *Atscc2-5* and *Atscc2-5*; *AtACT7*::*AtSCC2* transgenic plant. Bar = 3 cm. (C) The first 6 siliques of WT, *Atscc2-5* and *Atscc2-5*; *AtACT7*::*AtSCC2* transgenic plant. Bar = 1 cm. (D) The stripped siliques of WT, *Atscc2-5* and *Atscc2-5*; *AtACT7*::*AtSCC2* transgenic plant. Bar = 1 mm. (E) Western blotting by Flag antibody in WT, *Atscc2-5* homozygote, *Atscc2-5*; *AtACT7*::*AtSCC2* transgenic plant and *Atscc2-5* heterozygote plant. (F) Pollens of WT and *Atscc2-5*; *AtACT7*::*AtSCC2* transgenic plant stained by Alexander dye. Bar = 100 μm. (G) Chromosome spreads of pachytene and metaphase I in WT and *Atscc2-5*; *AtACT7*::*AtSCC2* transgenic plant meiocytes, hybridized with centromere probe and stained by DAPI. Bar = 5 μm.
(TIF)

S4 Fig The *AtSCC2* transcript is incorrectly spliced by 47 bp-deletion in *Atscc2-5* mutant. (A) The schematic diagram of normal and spliced *AtSCC2* transcript structure. (B) Nucleic acid electrophoresis of PCR products amplified by P7 primer in WT and *Atscc2-5*. Yellow arrows indicate the full length *AtSCC2* transcripts in *Atscc2-5* leaf and meiocyte, respectively. (C) Expression level of *AtSCC2* in leaves and meiocytes of WT and *Atscc2-5* mutant. Values are means ± SD of three independent experiments (* P < 0.05, ** P < 0.01, the significance of *AtSCC2* gene expression in WT leaf Vs *Atscc2-5* leaf, WT meiocyte Vs *Atscc2-5* meiocyte, *Atscc2-5* meiocyte Vs *Atscc2-5* leaf, by two-tailed Student’s *t* test).
(TIF)

S5 Fig Incorrectly spliced *AtSCC2* transcript is predicted to encode a truncated protein in *Atscc2-5*. (A) RNA-seq data show the read distribution of the 3’ terminal *AtSCC2* mRNA in WT and *Atscc2-5*. Orange arrow indicates the incorrectly spliced *AtSCC2* transcriptional reads in *Atscc2-5* meiocytes. (B) The *AtSCC2* coding amino acid sequences in WT and *Atscc2-5*.
(TIF)

S6 Fig The chromosome behaviors of WT and three compound heterozygous mutants. Chromosome spreads of WT, *line88*, *line88*^-^/*Atscc2-1*^-^, *line88*^-^/*Atscc2-3*^-^ and *line88*^-^/*Atscc2-4*^-^ compound heterozygous mutant male meiocytes, hybridized with centromere probe and stained by DAPI from zygotene to tetrad stage. Yellow arrows indicate chromosomal fragments. Yellow digitals indicate the number of centromeres at metaphase I. Bar = 5 μm.
(TIF)

S7 Fig Genetic analyses of *AtSCC2* with *AtSYN1* and *AtWAPL1/AtWAPL2*. DAPI stained chromosome and FISH with a centromere probe at zygotene, pachytene, metaphase I, telophase I, prophase II and metaphase II in (A) WT, (B) *Atscc2-5*, (C) *Atsyn1*, (D) *Atsyn1 Atscc2-5*, (E) *Atwapl1-1 Atwapl2*, (F) *Atwapl1-1 Atwapl2 Atscc2-5*. Bar = 5 μm.
(TIF)

S8 Fig *AtSCC2* is indispensable for normal assembly of axial element and synaptonemal complex. (A) The distribution of AtASY1 from leptotene to diakinesis in WT and *Atscc2-5*. Bar = 5 μm. (B) The distribution of AtASY1 in *Atspo11-1-1* and *Atspo11-1-1 Atscc2-5* zygotene chromosomes. Bar = 5 μm. (C) The AtZYP1 signals in WT and *Atscc2-5* pachytene chromosomes. Bar = 5 μm.
(TIF)

S9 Fig The AtSCC2 N terminus interacts with AtSCC4 and the schematic diagram of *AtSCC4* and *AtDMC1*::*AtSCC4*^*RNAi*^ structure. (A) The truncated AtSCC2 N terminal protein was used in yeast two-hybrid assay. (B) Yeast two-hybrid of the AtSCC2 N terminus with AtSCC4. (C) Validation of the AtSCC2-AtSCC4 interaction by Bimolecular Fluorescence Complementation (BiFC). (D) The schematic diagram of AtSCC4 protein and its transcript. (E) The two *AtDMC1*::*AtSCC4*^*RNAi*^*-M* and *AtDMC1*::*AtSCC4*^*RNAi*^*-N* plasmids used for transgenic plants.
(TIF)

S10 Fig Analyses of the fertility and *AtSCC4* expression level in *AtDMC1*::*AtSCC4*^*RNAi*^ lines. (A) Alexander staining anthers of WT and 8 *AtDMC1*::*AtSCC4*^*RNAi*^ transgenic plant. Bar = 100 μm. (B) Plots of live seeds per silique in WT and 8 *AtDMC1*::*AtSCC4*^*RNAi*^ transgenic plants (* P < 0.05 or ** P < 0.01, the significance of reduced seed number in *AtSCC4*^*RNAi*^ transgenic plants versus WT, by two-tailed Student’s *t* test). (C) The *AtSCC4* gene expression level in WT, *AtDMC1*::*AtSCC4*^*RNAi*^*-N T2-102*, *T2-129*, *T2-144*, *T2-147*, *AtDMC1*::*AtSCC4*^*RNAi*^*-M T2-161*, *T2-169*, *T2-171* and *T2-180* male meiocytes. Data were mean ± SD (two times repeated, * P < 0.05 or ** P < 0.01, the significance of *AtSCC4* gene expression in transgenic plants was compared with WT by two-tailed Student’s *t* test).
(TIF)

S11 Fig Chromosome behaviors of male meiocytes of WT and 8 *AtDMC1*::*AtSCC4*^*RNAi*^ mutants. Chromosome spreads of WT, *T2-102*, *T2-129*, *T2-144*, *T2-147*, *T2-161*, *T2-169*, *T2-171* and *T2-180* transgenic plant meiocytes at pachytene, metaphase I and metaphase II stage. Chromosomes were hybridized with centromere probe and stained by DAPI. Bar = 5 μm.
(TIF)

S12 Fig *AtDMC1*::*AtSCC4*^*RNAi*^*-N T2-102* and *AtDMC1*::*AtSCC4*^*RNAi*^*-M T2-161* transgenic plants show normal female meiosis but abnormal embryo morphogenesis at globular stage. (A) Siliques and seeds of the reciprocal cross lines between WT and *AtSCC4*^*RNAi*^ (Scale bar = 1 mm). (B) Chromosome spreads of WT, *T2-102* and *T2-161* in transgenic plant female meiocytes from leptotene to telophase II (Bar = 5 μm). (C) The embryo morphogenesis at globular stage in WT, *T2-102* and *T2-161* transgenic plants (Bar = 5 μm).
(TIF)

S13 Fig The plant specific SCC2 PHD domain is highly conserved in land plants. (A) The schematic diagram of SCC2 protein structures in *Ashbya gossypii*, *Saccharomyces cerevisiae*, *Arabidopsis thaliana* and *Homo sapiens*. (B) Alignment of PHD domains in plants. (C) Amino acid sequence alignment of the AtSCC2 PHD domain with other PHD domains. Stars indicate conserved “cysteine” or “histidine” amino acids.
(TIF)

S14 Fig RT-qPCR analyses of the *AtSCC2* transcript in core inflorescences of *Atscc2-5*; *AtACT7*::*AtSCC2*, *Atscc2-5*; *AtACT7*::*AtSCC2PHDΔ*, *Atscc2-1*; *AtACT7*::*AtSCC2* and *Atscc2-1*; *ACT7*::*AtSCC2PHDΔ*, comparing with WT and *Atscc2-5*. (Values are means ± SD of three independent experiments. ** P < 0.01, the significance of *AtSCC2* gene expression in transgenic plants compared with WT or *Atscc2-5* mutant by two-tailed Student’s *t* test).
(TIF)

S15 Fig The predicted protein structure of WT, AtSCC2, truncated AtSCC2 and a proposed model showing the role of *AtSCC2* in meiosis. (A) The predicted full-length protein structure of AtSCC2. Purple indicates the PHD domain and yellow indicates Nipped_B domain at the C terminus. The AtSCC2 C terminus forms a hook-like structure. (B) The predicted AtSCC2 truncated protein structure *in Atscc2-5*. Purple indicates the PHD domain, yellow indicates Nipped_B domain and green indicates the extra translated amino acids at C terminus. The AtSCC2-5 C terminus has a severely attenuated hook-like structure. (C) In wild type, at pre-leptotene stage, meiosis-specific cohesins start to be gradually loaded onto duplicated sister chromatids in an AtSCC2-dependent manner. When cells enter into leptotene, meiosis-specific cohesins are fully localized on chromosomes. The AtSYN1 loading may be mediated by AtSCC2 PHD domain binding to histones. In *Atscc2-5*, the tiny AtSCC2 can still load some cohesin from preleptotene to leptotene. However, the reduced AtSYN1 localization in chromosome and centromere ultimately causes meiotic defects.
(TIF)

S1 Table The segregation ratios of different *Atscc2* single mutant alleles. In these four alleles of *Atscc2* (*Atscc2-1*, *Atscc2-3*, *Atscc2-4* and *Atscc2-5*), the ratio of heterozygotes and wild types followed a 2:1 segregation pattern [p (χ^2^) > 0.23 in each case].
(DOCX)

S2 Table The segregation ratio of three *Atscc2* compound heterozygous plants with their corresponding *Atscc2-5* heterozygous F1 plants. The ratio of compound heterozygous F1 plants of two independent alleles (*Atscc2-5*^-^/*Atscc2-1*^-^ and *Atscc2-5*^-^/*Atscc2-3*^-^) with their corresponding *Atscc2-5* heterozygous F1 plants is 1:1 (χ^2^ ≤ χ_0.05_^2^ = 3.84, chi-square test). The ratio of F1 compound heterozygous of *Atscc2-5*^-^/*Atscc2-4*^-^ with *Atscc2-5* heterozygous F1 plants is not consistent with 1:1 (χ^2^ = 4.02 > χ_0.05_^2^ = 3.84), probably due to the low population number or an incompletely penetrant embryonic lethal phenotype.
(DOCX)

S3 Table The seed number between *AtSCC4*^*RNAi*^ and WT from reciprocal crossing. (DOCX)

S4 Table Primers used in this study. (DOCX)
