# Supplementary figures and images for: The cohesin loader SCC2 contains a PHD finger that is required for meiosis in land plants
Source: PLoS Genet. 2020 Jun 9;16(6):e1008849. doi: 10.1371/journal.pgen.1008849 (PMC7304647; doi:10.1371/journal.pgen.1008849)

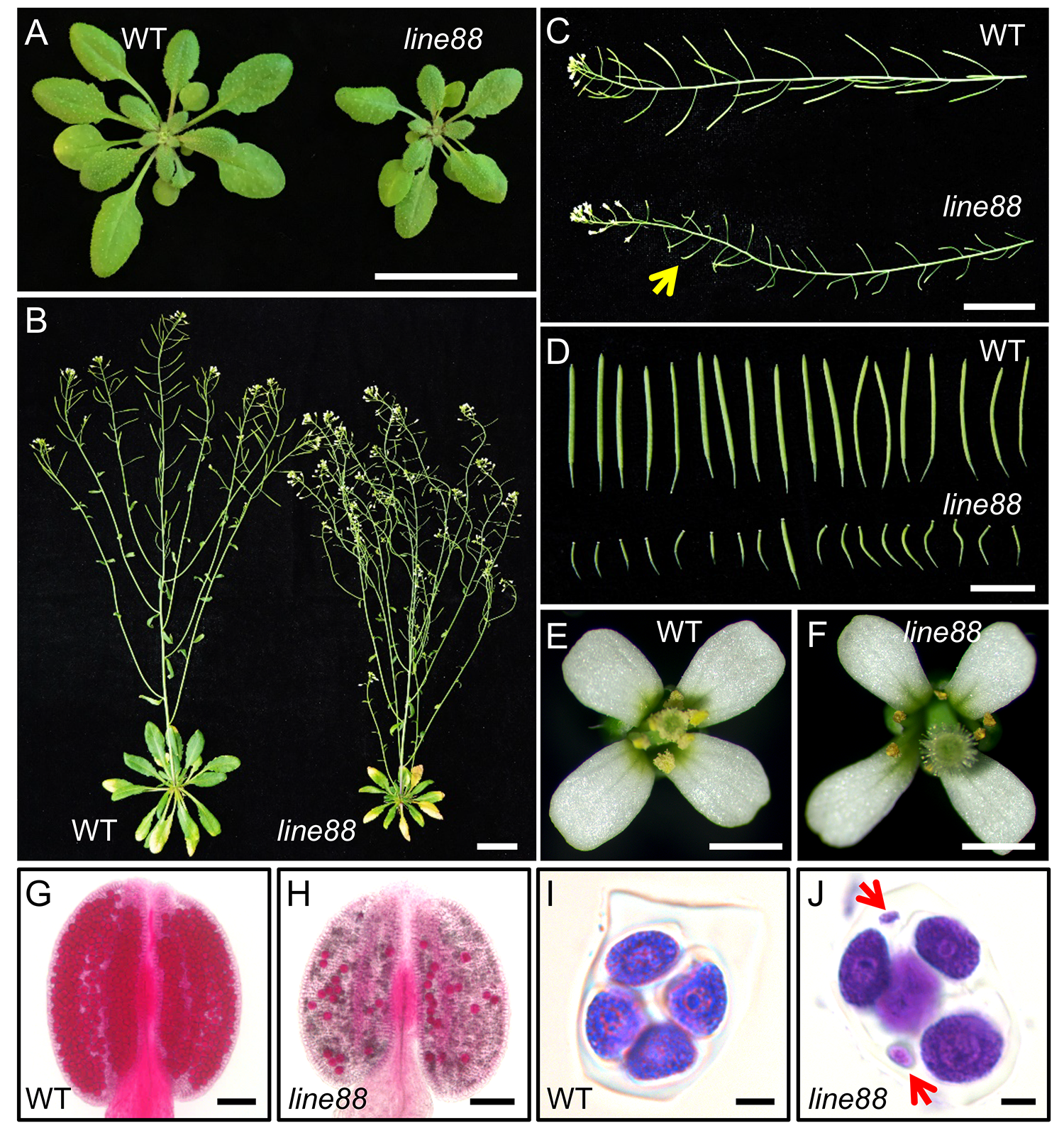

Supplement: S1 Fig — (A) The rosette leaves of four-week old WT and line88. Bar = 3 cm. (B) Comparison of an eight-week old WT plant and a line88 mutant plant. Bar = 3 cm. (C) Comparison of the WT and line88 stems. The yellow arrow indicates a short silique in line88. Bar = 3 cm. (D) The first 18 siliques of WT and line88. Bar = 1 cm. (E and F) The open flowers of WT and line88. Bar = 1 mm. (G and H) WT and line88 pollens stained with Alexander dye. Bar = 100 μm. (I and J) WT and line88 tetrads stained with Toluidine blue dye. Red arrows indicate the micronuclei. Bar = 5 μm. (TIF) [file pgen.1008849.s001.tif]

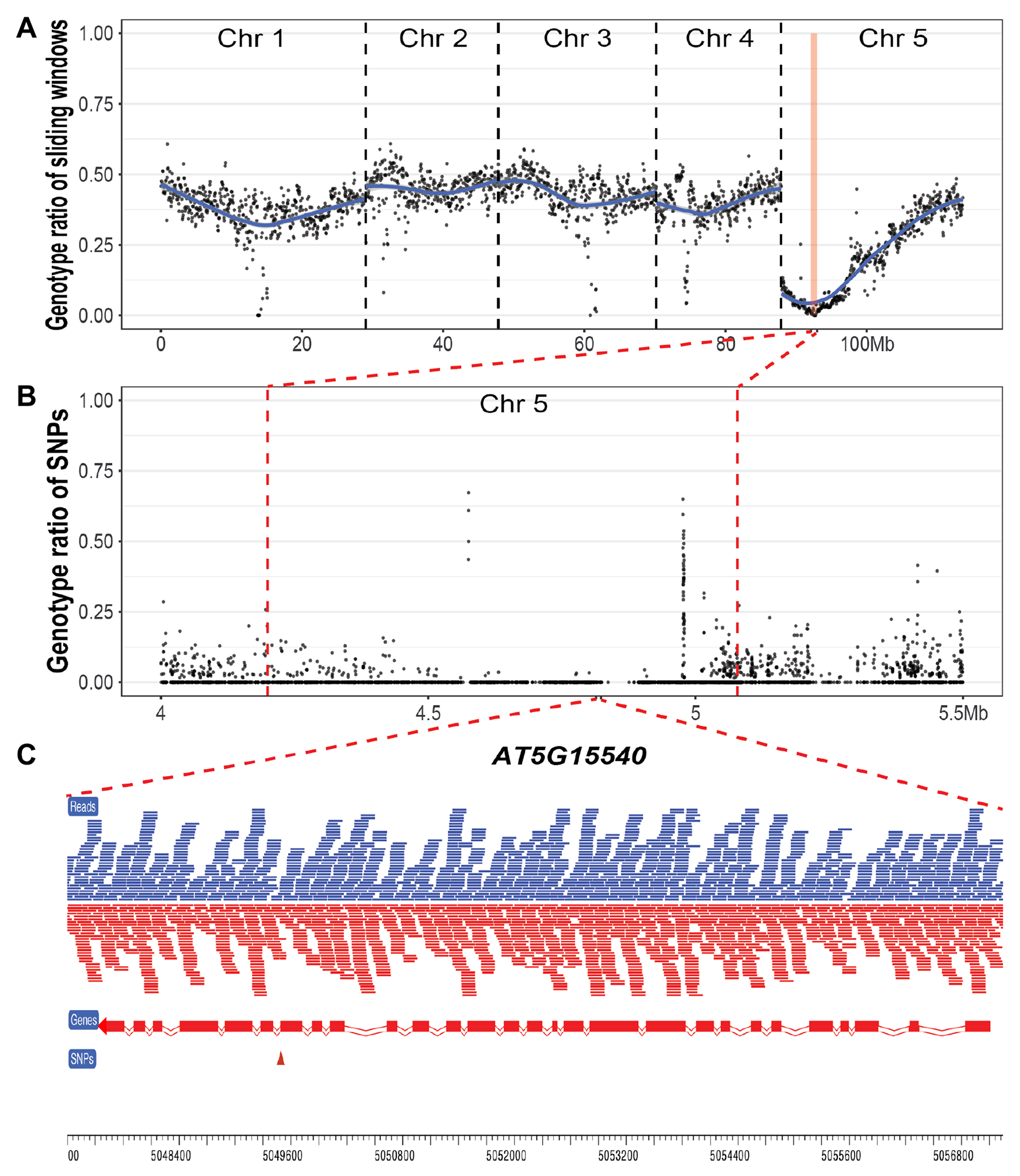

Supplement: S2 Fig — (A) Genotypic ratios are evaluated on sliding windows of 100-kb with step of 50-kb. The candidate genomic region is marked by a pink bar. (B) Distribution of genotypic ratio of SNPs in 4,403,200–5,324,800 bp on chromosome 5. (C) Mapping details of resequencing reads from line88 along with causal mutation (red triangle) on AT5G15540 gene. (TIF) [file pgen.1008849.s002.tif]

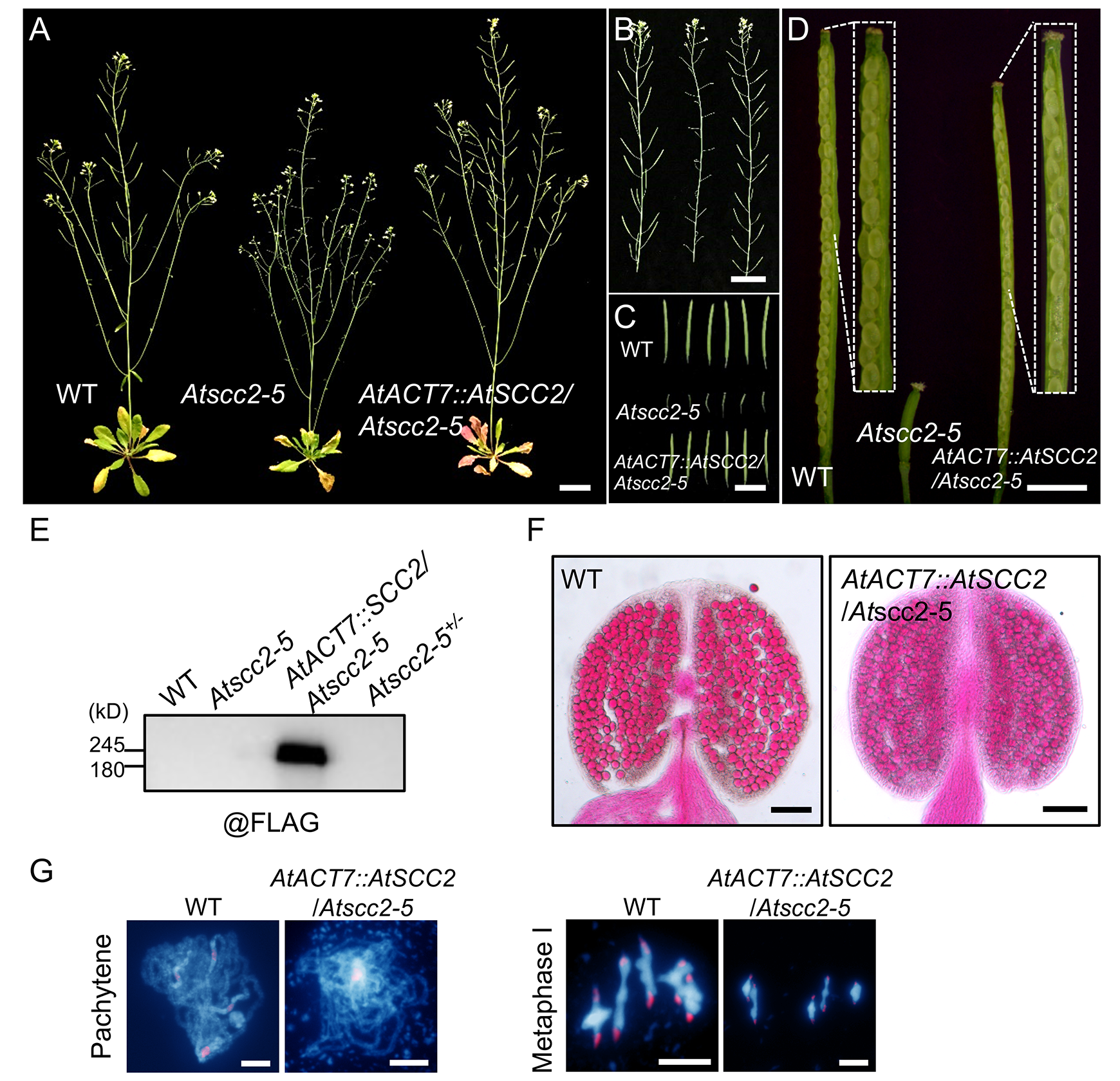

Supplement: S3 Fig — (A) Comparison of a WT plant, Atscc2-5 and Atscc2-5; AtACT7::AtSCC2 transgenic plant. Bar = 3 cm. (B) Comparison of the stems of WT, Atscc2-5 and Atscc2-5; AtACT7::AtSCC2 transgenic plant. Bar = 3 cm. (C) The first 6 siliques of WT, Atscc2-5 and Atscc2-5; AtACT7::AtSCC2 transgenic plant. Bar = 1 cm. (D) The stripped siliques of WT, Atscc2-5 and Atscc2-5; AtACT7::AtSCC2 transgenic plant. Bar = 1 mm. (E) Western blotting by Flag antibody in WT, Atscc2-5 homozygote, Atscc2-5; AtACT7::AtSCC2 transgenic plant and Atscc2-5 heterozygote plant. (F) Pollens of WT and Atscc2-5; AtACT7::AtSCC2 transgenic plant stained by Alexander dye. Bar = 100 μm. (G) Chromosome spreads of pachytene and metaphase I in WT and Atscc2-5; AtACT7::AtSCC2 transgenic plant meiocytes, hybridized with centromere probe and stained by DAPI. Bar = 5 μm. (TIF) [file pgen.1008849.s003.tif]

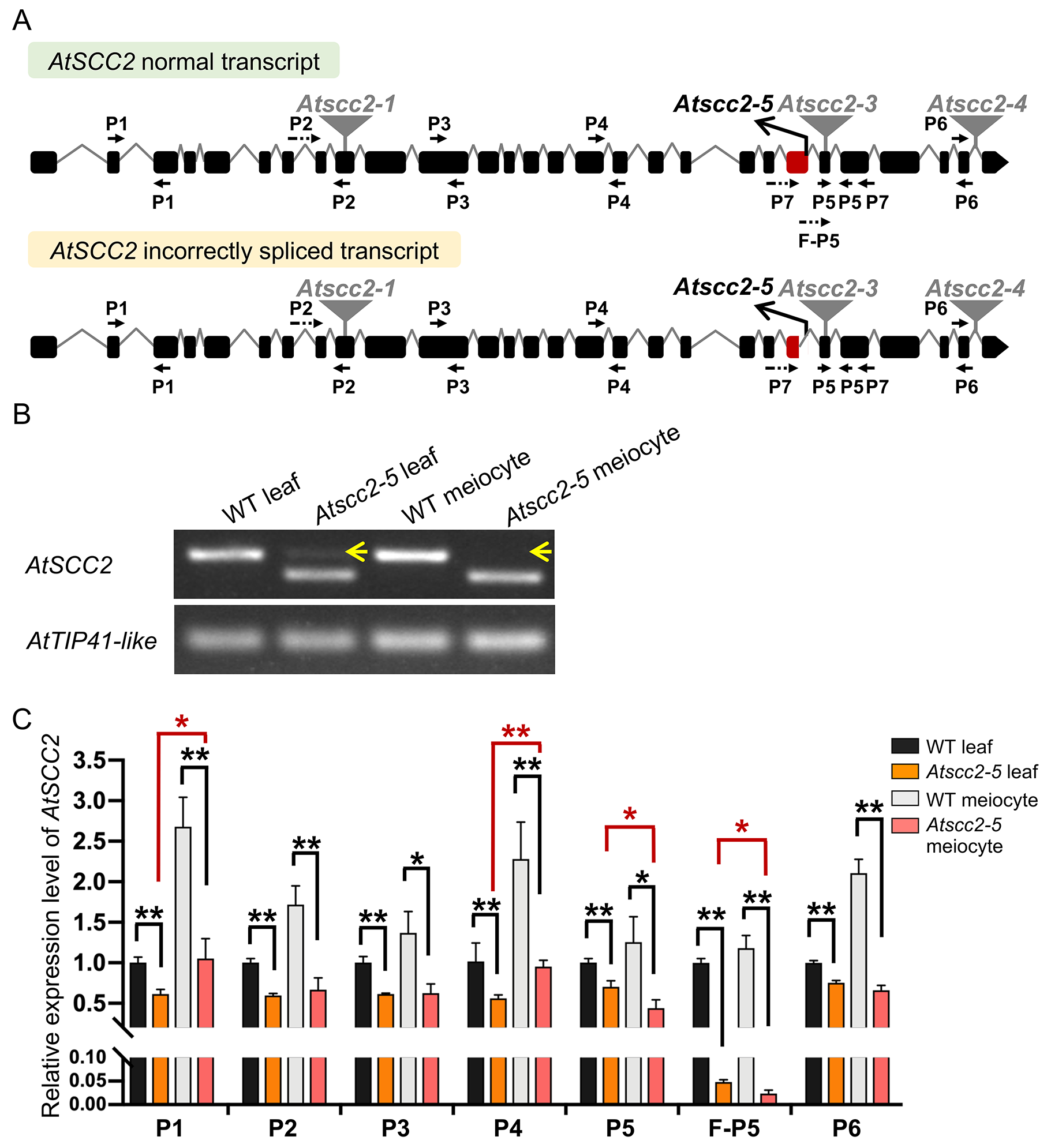

Supplement: S4 Fig — (A) The schematic diagram of normal and spliced AtSCC2 transcript structure. (B) Nucleic acid electrophoresis of PCR products amplified by P7 primer in WT and Atscc2-5. Yellow arrows indicate the full length AtSCC2 transcripts in Atscc2-5 leaf and meiocyte, respectively. (C) Expression level of AtSCC2 in leaves and meiocytes of WT and Atscc2-5 mutant. Values are means ± SD of three independent experiments (* P < 0.05, ** P < 0.01, the significance of AtSCC2 gene expression in WT leaf Vs Atscc2-5 leaf, WT meiocyte Vs Atscc2-5 meiocyte, Atscc2-5 meiocyte Vs Atscc2-5 leaf, by two-tailed Student’s t test). (TIF) [file pgen.1008849.s004.tif]

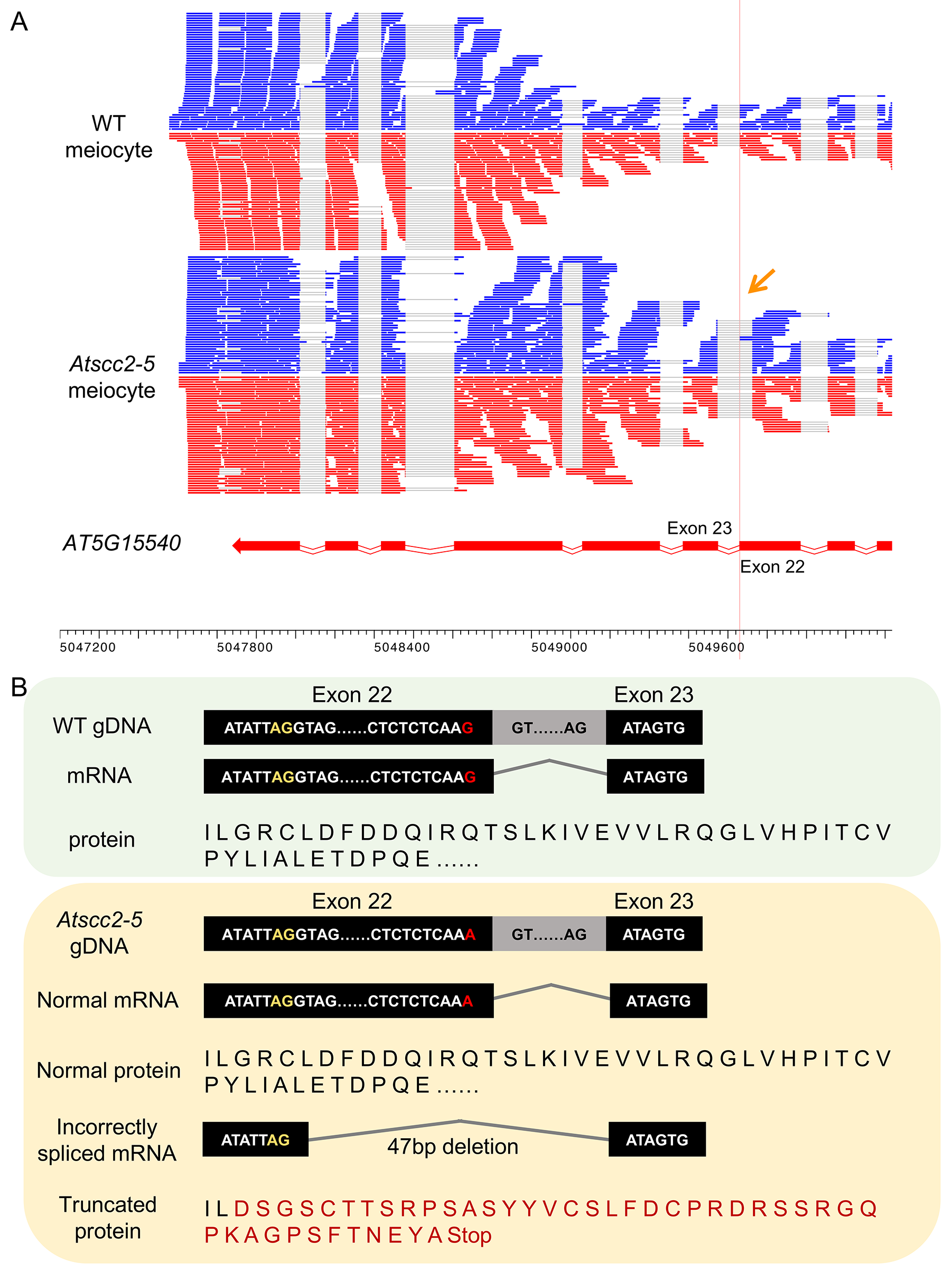

Supplement: S5 Fig — (A) RNA-seq data show the read distribution of the 3’ terminal AtSCC2 mRNA in WT and Atscc2-5. Orange arrow indicates the incorrectly spliced AtSCC2 transcriptional reads in Atscc2-5 meiocytes. (B) The AtSCC2 coding amino acid sequences in WT and Atscc2-5. (TIF) [file pgen.1008849.s005.tif]

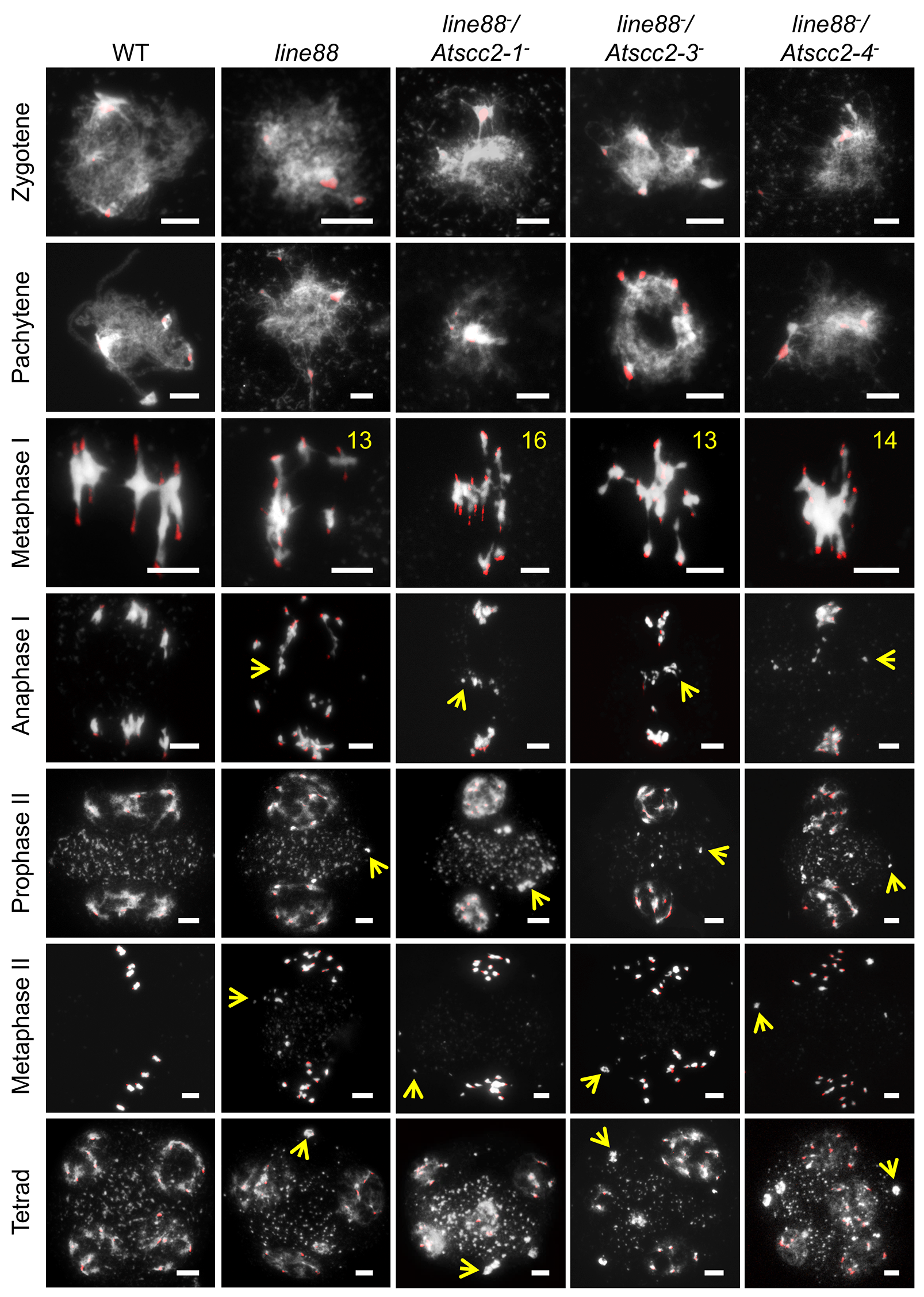

Supplement: S6 Fig — Chromosome spreads of WT, line88, line88-/Atscc2-1-, line88-/Atscc2-3- and line88-/Atscc2-4- compound heterozygous mutant male meiocytes, hybridized with centromere probe and stained by DAPI from zygotene to tetrad stage. Yellow arrows indicate chromosomal fragments. Yellow digitals indicate the number of centromeres at metaphase I. Bar = 5 μm. (TIF) [file pgen.1008849.s006.tif]

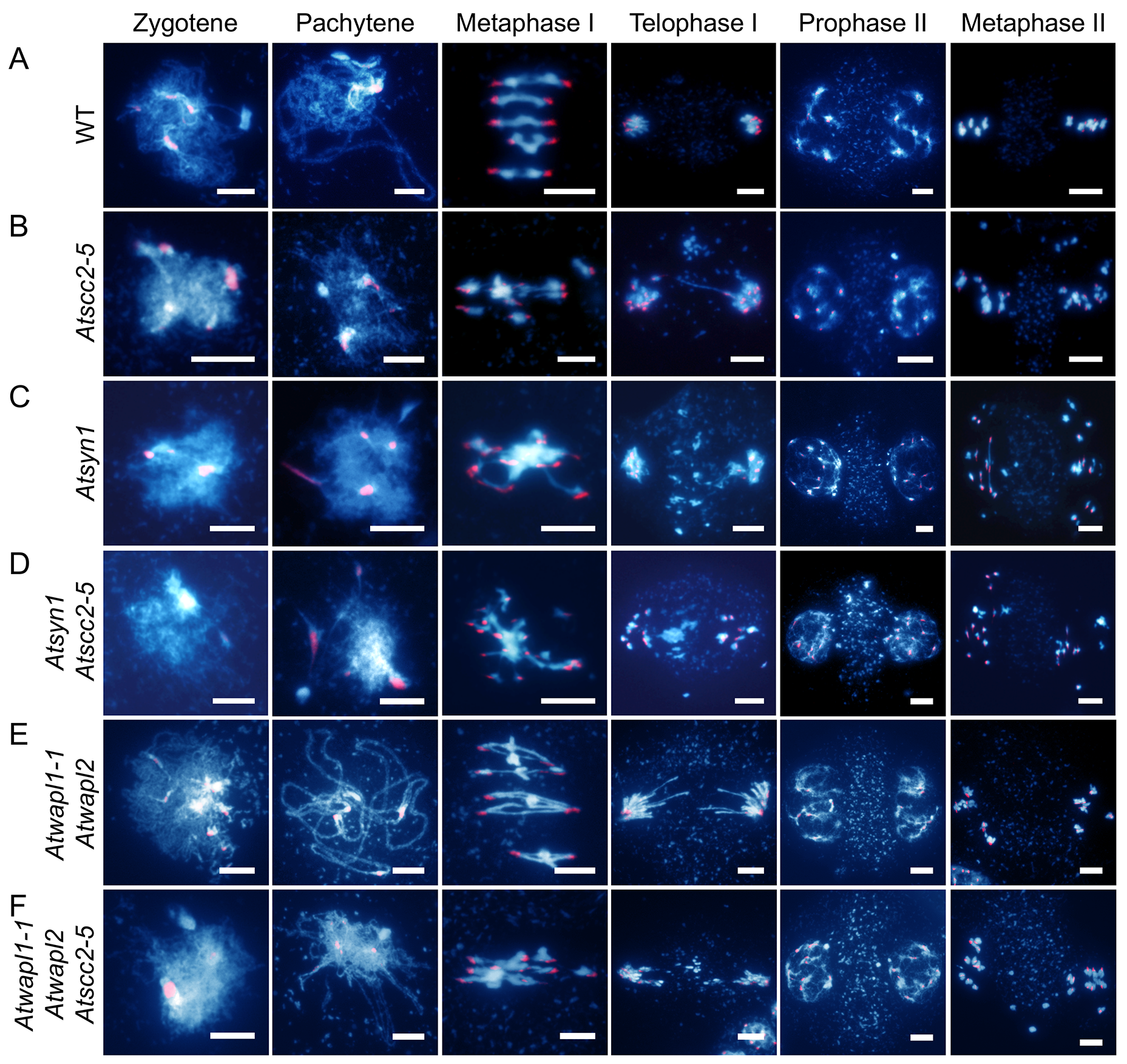

Supplement: S7 Fig — DAPI stained chromosome and FISH with a centromere probe at zygotene, pachytene, metaphase I, telophase I, prophase II and metaphase II in (A) WT, (B) Atscc2-5, (C) Atsyn1, (D) Atsyn1 Atscc2-5, (E) Atwapl1-1 Atwapl2, (F) Atwapl1-1 Atwapl2 Atscc2-5. Bar = 5 μm. (TIF) [file pgen.1008849.s007.tif]

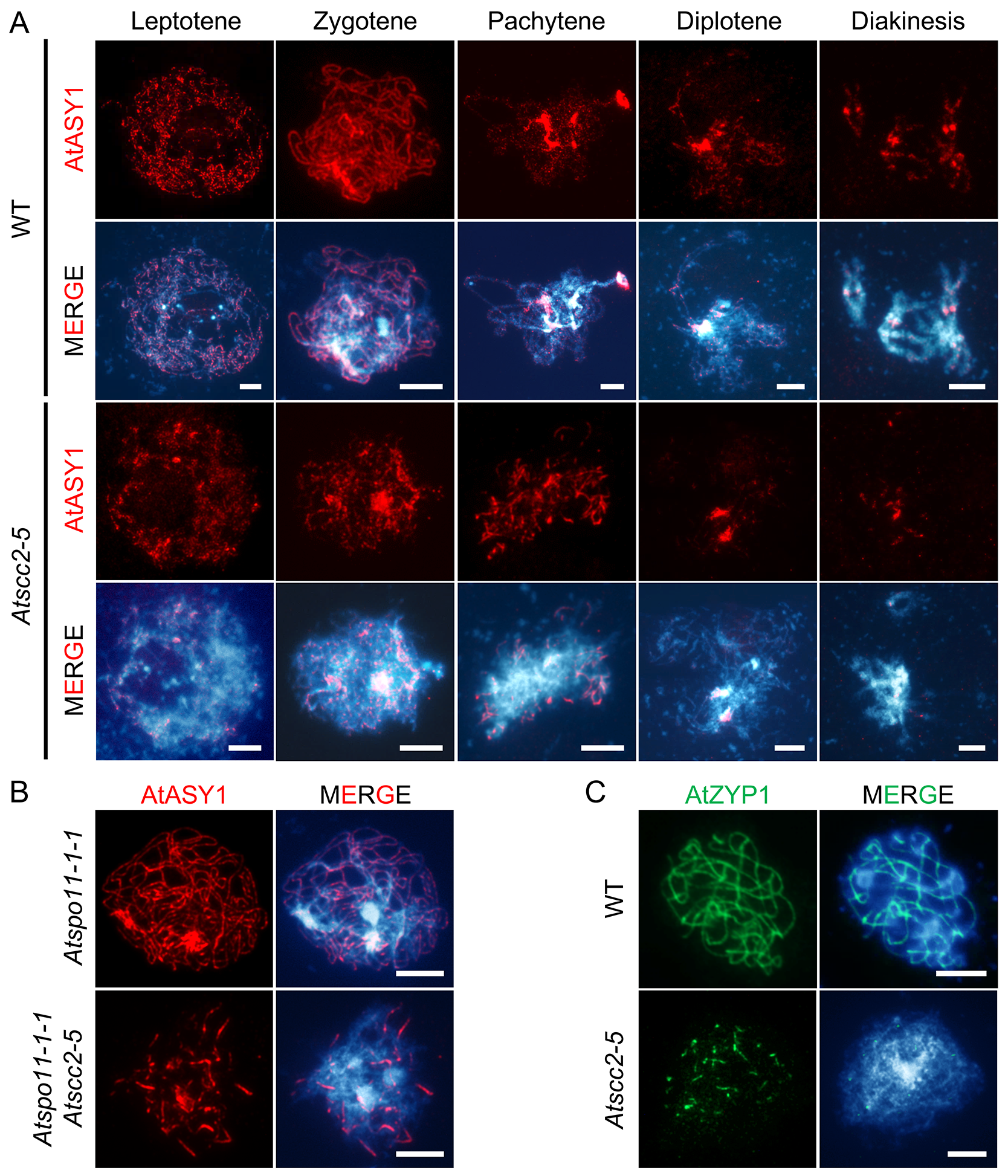

Supplement: S8 Fig — (A) The distribution of AtASY1 from leptotene to diakinesis in WT and Atscc2-5. Bar = 5 μm. (B) The distribution of AtASY1 in Atspo11-1-1 and Atspo11-1-1 Atscc2-5 zygotene chromosomes. Bar = 5 μm. (C) The AtZYP1 signals in WT and Atscc2-5 pachytene chromosomes. Bar = 5 μm. (TIF) [file pgen.1008849.s008.tif]

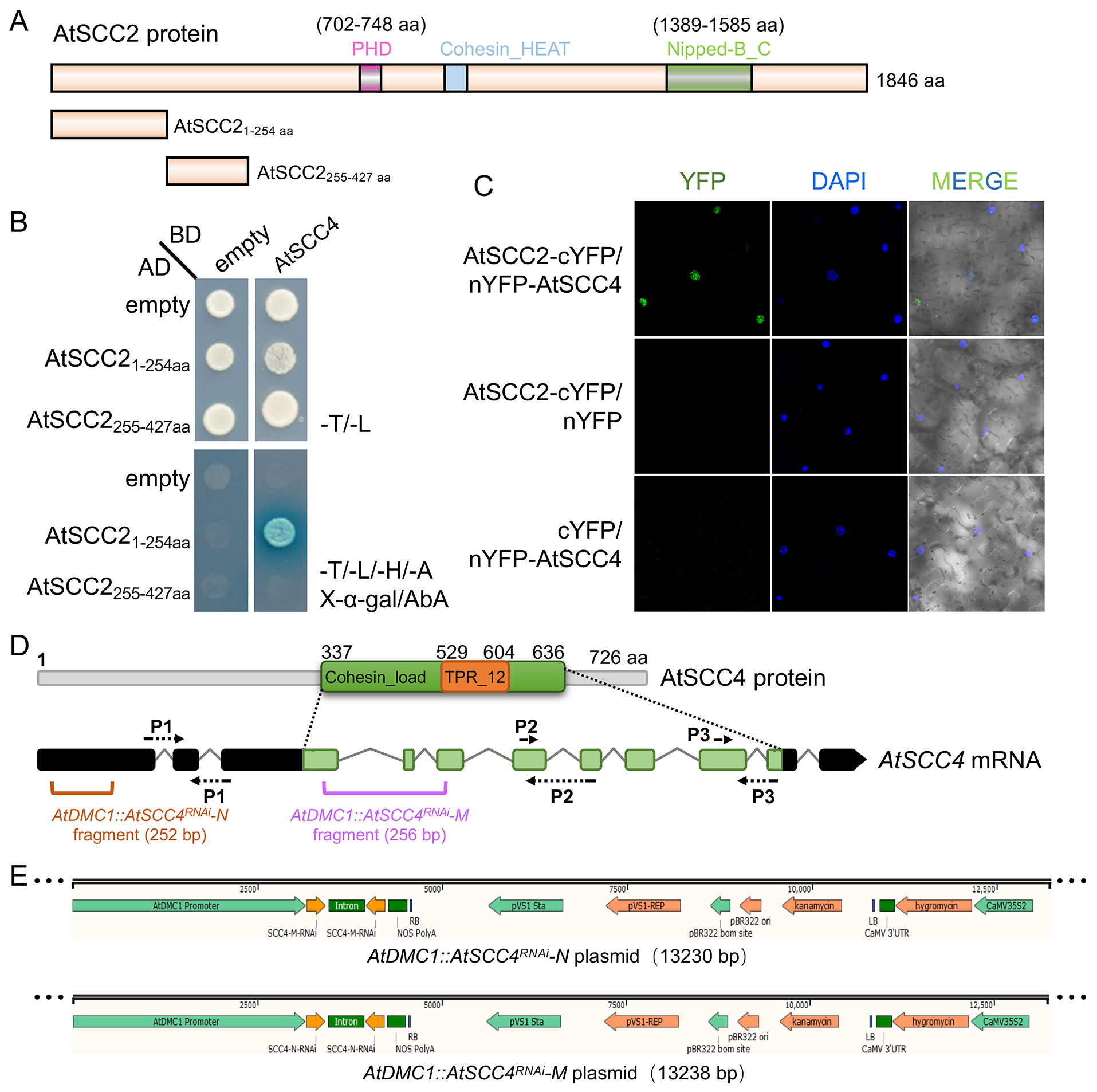

Supplement: S9 Fig — (A) The truncated AtSCC2 N terminal protein was used in yeast two-hybrid assay. (B) Yeast two-hybrid of the AtSCC2 N terminus with AtSCC4. (C) Validation of the AtSCC2-AtSCC4 interaction by Bimolecular Fluorescence Complementation (BiFC). (D) The schematic diagram of AtSCC4 protein and its transcript. (E) The two AtDMC1::AtSCC4RNAi-M and AtDMC1::AtSCC4RNAi-N plasmids used for transgenic plants. (TIF) [file pgen.1008849.s009.tif]

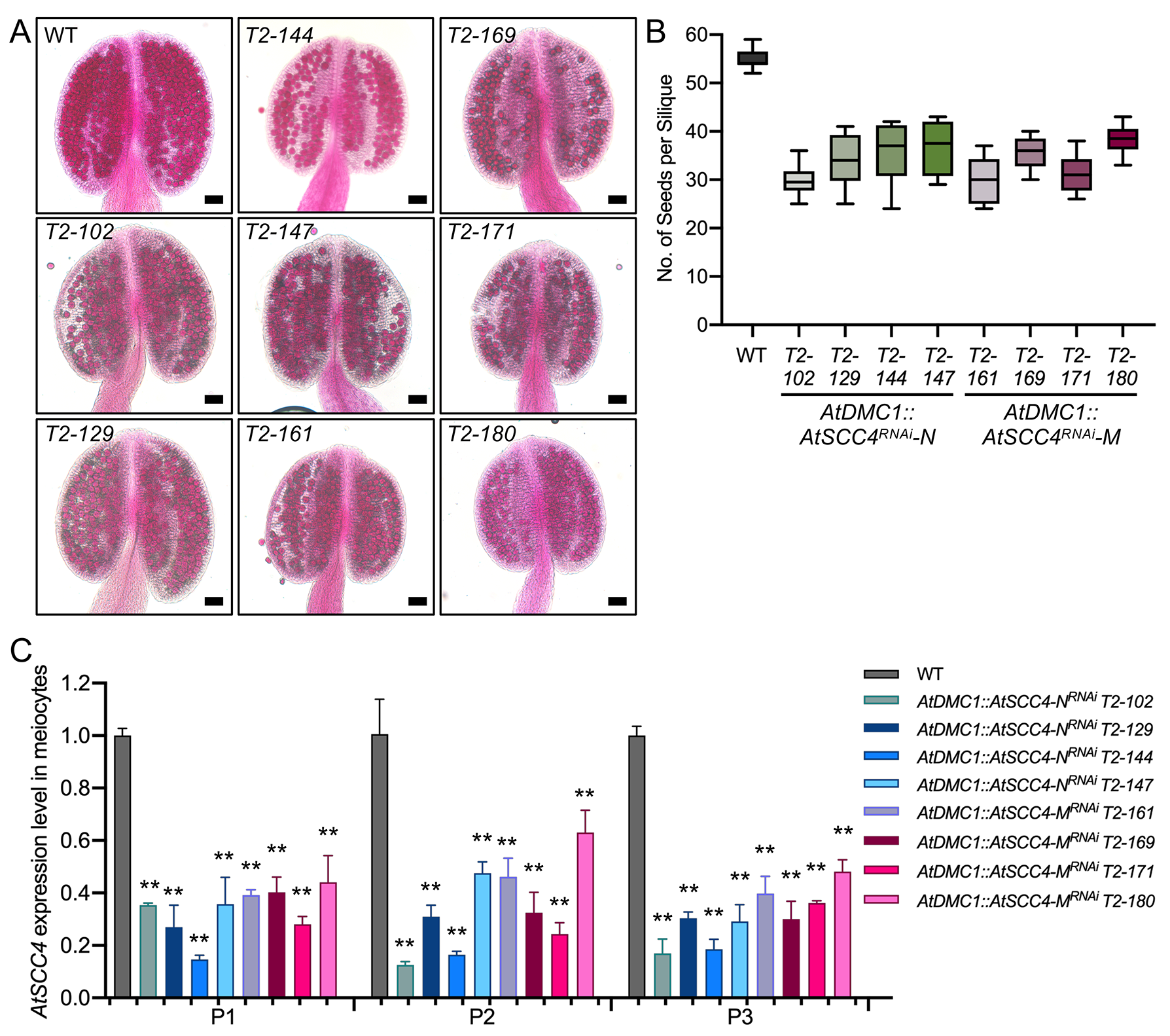

Supplement: S10 Fig — (A) Alexander staining anthers of WT and 8 AtDMC1::AtSCC4RNAi transgenic plant. Bar = 100 μm. (B) Plots of live seeds per silique in WT and 8 AtDMC1::AtSCC4RNAi transgenic plants (* P < 0.05 or ** P < 0.01, the significance of reduced seed number in AtSCC4RNAi transgenic plants versus WT, by two-tailed Student’s t test). (C) The AtSCC4 gene expression level in WT, AtDMC1::AtSCC4RNAi-N T2-102, T2-129, T2-144, T2-147, AtDMC1::AtSCC4RNAi-M T2-161, T2-169, T2-171 and T2-180 male meiocytes. Data were mean ± SD (two times repeated, * P < 0.05 or ** P < 0.01, the significance of AtSCC4 gene expression in transgenic plants was compared with WT by two-tailed Student’s t test). (TIF) [file pgen.1008849.s010.tif]

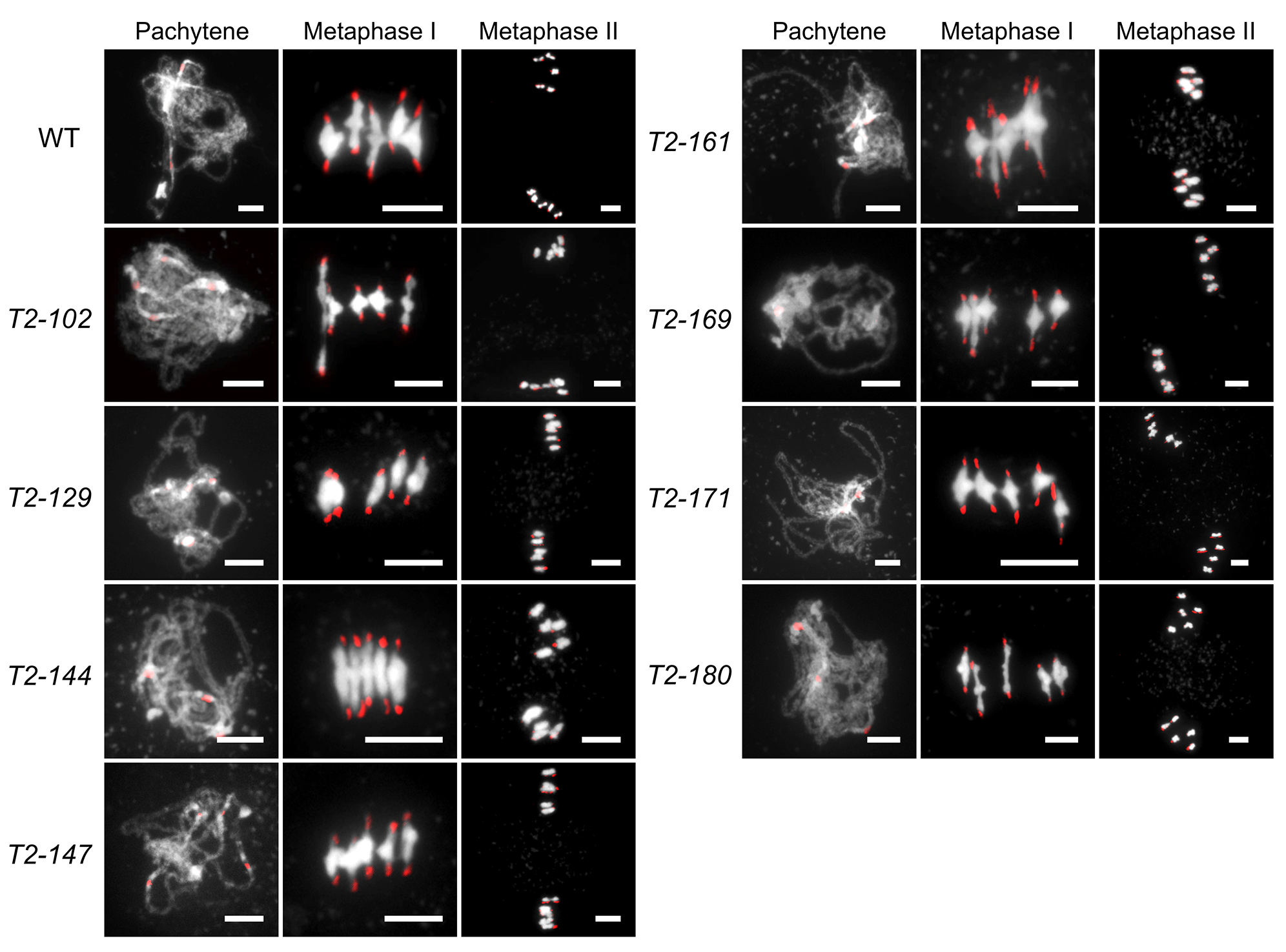

Supplement: S11 Fig — Chromosome spreads of WT, T2-102, T2-129, T2-144, T2-147, T2-161, T2-169, T2-171 and T2-180 transgenic plant meiocytes at pachytene, metaphase I and metaphase II stage. Chromosomes were hybridized with centromere probe and stained by DAPI. Bar = 5 μm. (TIF) [file pgen.1008849.s011.tif]

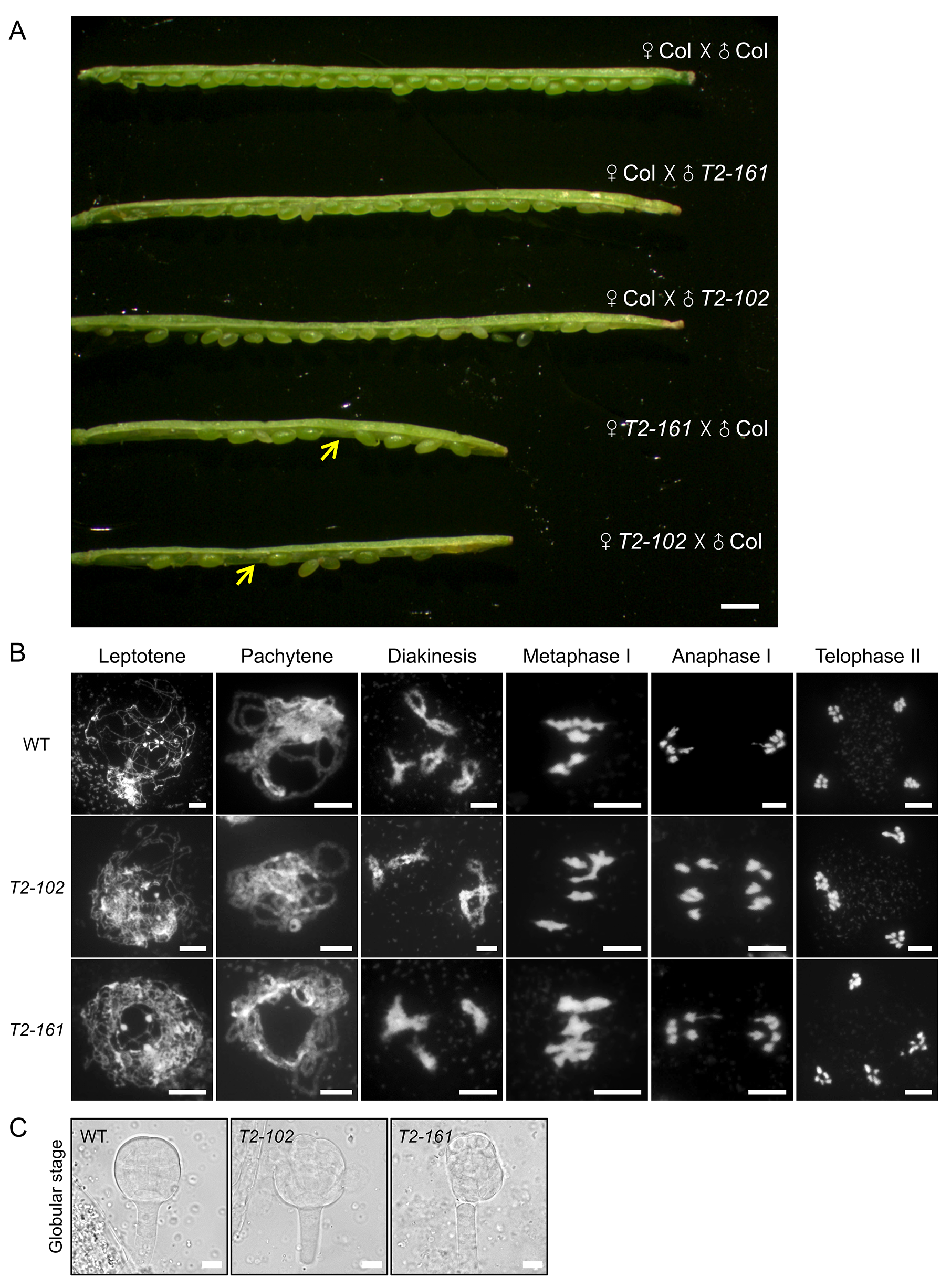

Supplement: S12 Fig — (A) Siliques and seeds of the reciprocal cross lines between WT and AtSCC4RNAi (Scale bar = 1 mm). (B) Chromosome spreads of WT, T2-102 and T2-161 in transgenic plant female meiocytes from leptotene to telophase II (Bar = 5 μm). (C) The embryo morphogenesis at globular stage in WT, T2-102 and T2-161 transgenic plants (Bar = 5 μm). (TIF) [file pgen.1008849.s012.tif]

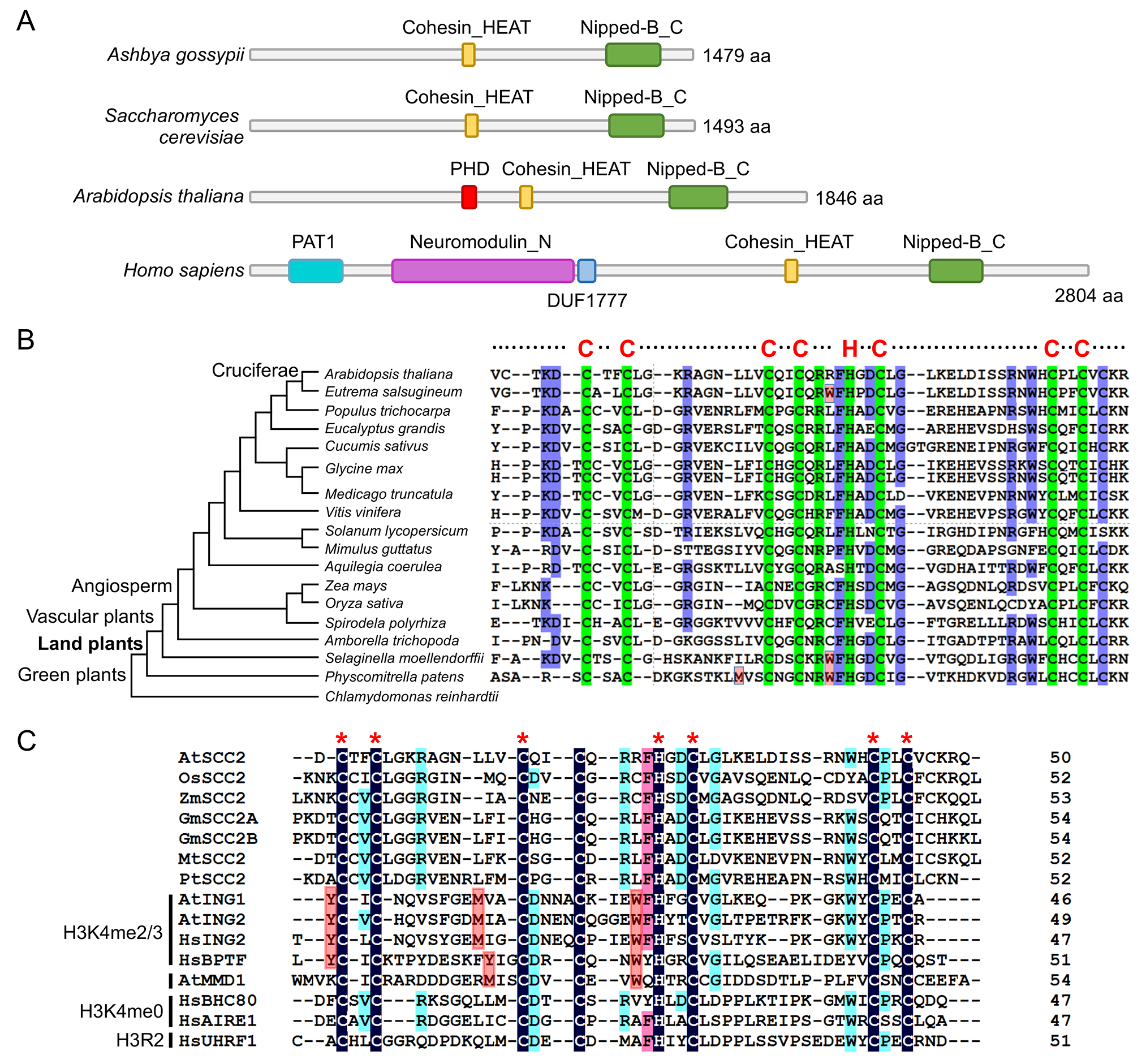

Supplement: S13 Fig — (A) The schematic diagram of SCC2 protein structures in Ashbya gossypii, Saccharomyces cerevisiae, Arabidopsis thaliana and Homo sapiens. (B) Alignment of PHD domains in plants. (C) Amino acid sequence alignment of the AtSCC2 PHD domain with other PHD domains. Stars indicate conserved “cysteine” or “histidine” amino acids. (TIF) [file pgen.1008849.s013.tif]

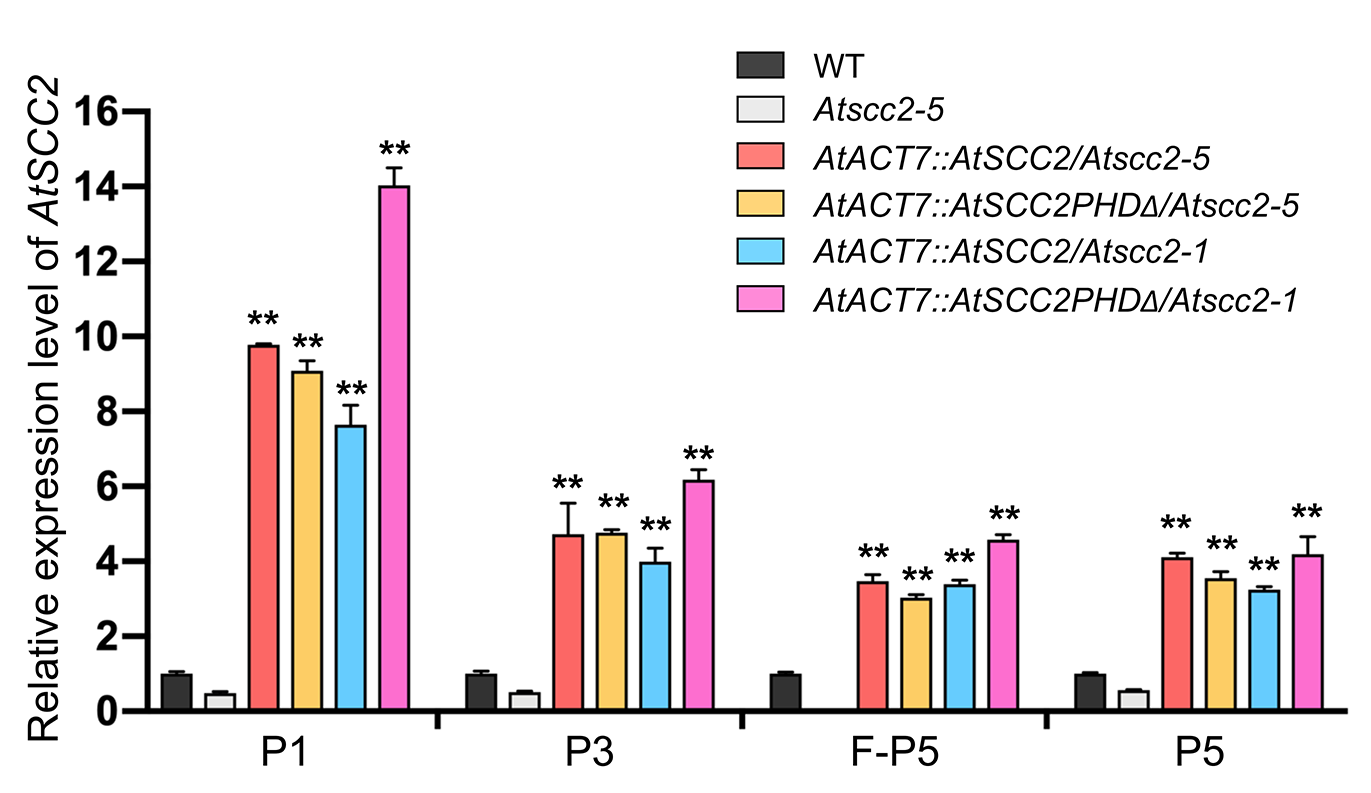

Supplement: S14 Fig — (Values are means ± SD of three independent experiments. ** P < 0.01, the significance of AtSCC2 gene expression in transgenic plants compared with WT or Atscc2-5 mutant by two-tailed Student’s t test). (TIF) [file pgen.1008849.s014.tif]

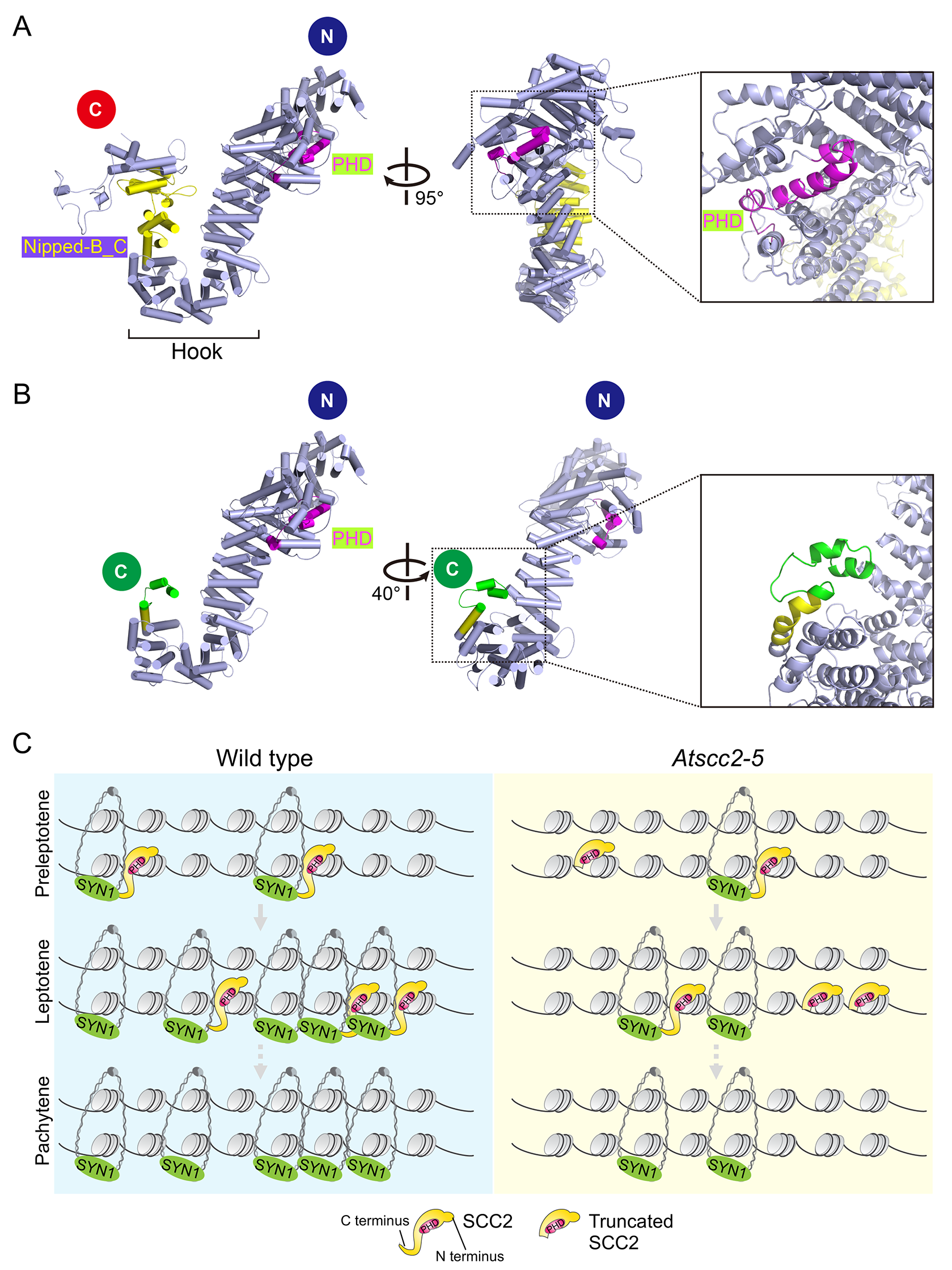

Supplement: S15 Fig — (A) The predicted full-length protein structure of AtSCC2. Purple indicates the PHD domain and yellow indicates Nipped_B domain at the C terminus. The AtSCC2 C terminus forms a hook-like structure. (B) The predicted AtSCC2 truncated protein structure in Atscc2-5. Purple indicates the PHD domain, yellow indicates Nipped_B domain and green indicates the extra translated amino acids at C terminus. The AtSCC2-5 C terminus has a severely attenuated hook-like structure. (C) In wild type, at pre-leptotene stage, meiosis-specific cohesins start to be gradually loaded onto duplicated sister chromatids in an AtSCC2-dependent manner. When cells enter into leptotene, meiosis-specific cohesins are fully localized on chromosomes. The AtSYN1 loading may be mediated by AtSCC2 PHD domain binding to histones. In Atscc2-5, the tiny AtSCC2 can still load some cohesin from preleptotene to leptotene. However, the reduced AtSYN1 localization in chromosome and centromere ultimately causes meiotic defects. (TIF) [file pgen.1008849.s015.tif]
