# Supplementary material for: The cohesin loader SCC2 contains a PHD finger that is required for meiosis in land plants
Source: PLoS Genet. 2020 Jun 9;16(6):e1008849. doi: 10.1371/journal.pgen.1008849 (PMC7304647; doi:10.1371/journal.pgen.1008849)
Supplement: S1 Table — In these four alleles of Atscc2 (Atscc2-1, Atscc2-3, Atscc2-4 and Atscc2-5), the ratio of heterozygotes and wild types followed a 2:1 segregation pattern [p (χ2) > 0.23 in each case]. (DOCX) [file pgen.1008849.s016.docx]

**S1 Table. The segregation ratios of different *Atscc2* single mutant alleles**

|  | **F2 generation** | | |
| --- | --- | --- | --- |
| **Genotype** | **Wild type** | **Heterozygote** | **Homozygote** |
| *Atscc2-1* | 67 | 110 | 0 |
| *Atscc2-3* | 33 | 63 | 0 |
| *Atscc2-4* | 33 | 58 | 0 |
| *Atscc2-5* | 409 | 816 | 278 |

In these four alleles of *Atscc2* (*Atscc2-1*, *Atscc2-3*, *Atscc2-4* and *Atscc2-5*), the ratio of heterozygotes and wild types followed a 2:1 segregation pattern [p (χ^2^) > 0.23 in each case].
