# Supplementary material for: The cohesin loader SCC2 contains a PHD finger that is required for meiosis in land plants
Source: PLoS Genet. 2020 Jun 9;16(6):e1008849. doi: 10.1371/journal.pgen.1008849 (PMC7304647; doi:10.1371/journal.pgen.1008849)
Supplement: S2 Table — The ratio of compound heterozygous F1 plants of two independent alleles (Atscc2-5-/Atscc2-1- and Atscc2-5-/Atscc2-3-) with their corresponding Atscc2-5 heterozygous F1 plants is 1:1 (χ2 ≤ χ0.052 = 3.84, chi-square test). The ratio of F1 compound heterozygous of Atscc2-5-/Atscc2-4- with Atscc2-5 heterozygous F1 plants is not consistent with 1:1 (χ2 = 4.02 > χ0.052 = 3.84), probably due to the low population number or an incompletely penetrant embryonic lethal phenotype. (DOCX) [file pgen.1008849.s017.docx]

**S2 Table. The segregation ratios of three *Atscc2* compound heterozygous plants with their corresponding *Atscc2-5* heterozygous F1 plants**

| **Genotype** | **Compound heterozygous F1 plants** | ***Atscc2-5* heterozygous F1 plants** |
| --- | --- | --- |
| *Atscc2-5^-^/Atscc2-1^-^* | 15 | 29 |
| *Atscc2-5^-^/Atscc2-3^-^* | 20 | 32 |
| *Atscc2-5^-^/Atscc2-4^-^* | 20 | 36 |

The ratio of compound heterozygous F1 plants of two independent alleles (*Atscc2-5^-^*/*Atscc2-1^-^* and *Atscc2-5^-^*/*Atscc2-3^-^*) with their corresponding *Atscc2-5* heterozygous F1 plants is 1:1 (χ^2^ ≤ χ_0.05_^2^ = 3.84, chi-square test). The ratio of F1 compound heterozygous of *Atscc2-5^-^*/*Atscc2-4^-^* with *Atscc2-5* heterozygous F1 plants is not consistent with 1:1 (χ^2^ = 4.02 > χ_0.05_^2^ = 3.84), probably due to the low population number or an incompletely penetrant embryonic lethal phenotype.
