# Supplementary material for: The cohesin loader SCC2 contains a PHD finger that is required for meiosis in land plants
Source: PLoS Genet. 2020 Jun 9;16(6):e1008849. doi: 10.1371/journal.pgen.1008849 (PMC7304647; doi:10.1371/journal.pgen.1008849)
Supplement: S3 Table — (DOCX) [file pgen.1008849.s018.docx]

| **♀ × ♂** | **Normal seeds**  **(per silique)** | **Abnormal seeds**  **(per silique)** | **Total seeds**  **(per silique)** |
| --- | --- | --- | --- |
| **Col × Col**  **(n = 15)** | 45.8 ± 3.0 | 0.0 ± 0.0 | 45.8 ± 3.0 |
| **Col × *AtDMC1::AtSCC4-M^RNAi^ T2-161* (n = 6)** | 39.8 ± 5.7 | 4.5 ± 3.9 | 44.3 ± 2.4 |
| **Col × *AtDMC1::AtSCC4-N^RNAi^ T2-102* (n = 11)** | 35.5 ± 6.4 | 3.6 ± 2.5 | 39.2 ± 5.8 |
| ***AtDMC1::AtSCC4-M^RNAi^ T2-161* × Col (n = 13)** | 15.9 ± 4.2 | 17.0 ± 3.7 | 32.9 ± 4.4 |
| ***AtDMC1::AtSCC4-N^RNAi^ T2-102* × Col (n = 12)** | 14.6 ± 3.6 | 15.9 ± 4.6 | 30.5 ± 4.6 |

**S3 Table. The seed number between *AtSCC4^RNAi^* and WT from reciprocal crossing**
