# Supplementary material for: The cohesin loader SCC2 contains a PHD finger that is required for meiosis in land plants
Source: PLoS Genet. 2020 Jun 9;16(6):e1008849. doi: 10.1371/journal.pgen.1008849 (PMC7304647; doi:10.1371/journal.pgen.1008849)
Supplement: S4 Table — (DOCX) [file pgen.1008849.s019.docx]

**S4 Table. Primers used in this study**

| **Primer** | **sequence** |
| --- | --- |
| *AtTIP41-like*-RT-F | GTGAAAACTGTTGGAGAGAAGCAA |
| *AtTIP41-like*-RT-R | TCAACTGGATACCCTTTCGCA |
| *AtSCC2*-RT-P1-F | GCAATCCTGGAGCATTTGAG |
| *AtSCC2*-RT-P1-R | CGCTGCATCTTGACAGGAAC |
| *AtSCC2*-RT-P2-F | GCGTCAGTAAACAGGATCTC |
| *AtSCC2*-RT-P2-R | ACCAACAACAGGTCCTTGAG |
| *AtSCC2*-RT-P3-F | ACACCAAGGGTACTGGTAAG |
| *AtSCC2*-RT-P3-R | GCTGATCCTACATCCTGAAG |
| *AtSCC2*-RT-P4-F | GCTGATGTGCAAGTGCTTAC |
| *AtSCC2*-RT-P4-R | AGGATCCACCAGACAGAAAG |
| *AtSCC2*-RT-P5-F | GGCCTAGTGCATCCTATTAC |
| *AtSCC2*-RT-F-P5-F | CCAGACCTCTCTCAAGATAG |
| *AtSCC2*-RT-S-P5-F | GGGATAAAATATTAGATAGTGGAAGTTG |
| *AtSCC2*-RT-P5-R | AAGCCGGATACTTCTCATGC |
| *AtSCC2*-RT-P6-F | CTCCAACGGAACCATTGAAG |
| *AtSCC2*-RT-P6-R | GGTAAGTCAGTGCGAGTTTC |
| *AtSCC2*-AGE-P7-F | GGGCGTATGAAGATGCAAGC |
| *AtSCC2*-AGE-P7-R | GTTGGCCTCTTGAGGATCTG |
| *Atscc2-5*-genotyping-F | GTGCTAGCTGTTGCACATTC |
| *Atscc2-5*-genotyping-R | TTTCGAGAAACCCGGTTTCC |
| *Atscc2-5*-pmG-F | CGCCAGACCTCTCTCAcG |
| *Atscc2-5*-pmA-F | CGCCAGACCTCTCTCAcA |
| *Atscc2-1*-genotyping-F | TCAACCCTGCATTATGTAGCAG |
| *Atscc2-1*-genotyping-R | ATGTAAGCGCAAAAATTGTGC |
| *Atscc2-3*-genotyping-F | TTTAAACGACATCTCCGAACG |
| *Atscc2-3*-genotyping-R | TTACTGAGCCATTCCAGGTTG |
| *Atscc2-4*-genotyping-F | ATGGCCATGCAACTCTAATTG |
| *Atscc2-4*-genotyping-R | TTACGAGCCTAAACAACAGCG |
| *Atspo11-1-1*-genotyping-F | CCCTTTGGTTTATCAGAGCTGC |
| *Atspo11-1-1*-genotyping-R | CCAACGGCCCAACGTGTTTA |
| LB5 | ACTGGGATTCGTCTTGGACA |
| *Atatm-2*-genotyping-F | ATCCATGTGGTTCAGTCTTGC |
| *Atatm-2*-genotyping-R | TTGGTATCCTGCAGAGGAAAG |
| LBb1.3 | ATTTTGCCGATTTCGGAAC |
| *Atdmc1*-genotyping-F | TAGACGGATGATAACTATGACG |
| *Atdmc1*-genotyping-R | ACCCAGCGATTAAGCAACAA |
| DS 3' | CCGGTATATCCCGTTTTCG |
| *Atrad51-1*-genotyping-F | CGGTAGCACTATCGACAATC |
| *Atrad51-1*-genotyping-R | GTGATGCCAAGGTTGACAAG |
| LBGA | ATATTGACCATCATACTCATTGC |
| *Atmsh4-1*-genotyping-F | TTCCCTTCTTGCAGGTTGTG |
| *Atmsh4-1*-genotyping-R | CATATGCGCTTCCAGATACC |
| *Atmus81-2*-genotyping-F | TGGTGAAATCTAGCAACCCAG |
| *Atmus81-2*-genotyping-R | AATTTTCCACAAACCCTTTGG |
| *Atsyn1*-genotyping-F | TTGTCCGGTATGTTTTGTTCC |
| *Atsyn1*-genotyping-R | ACGTGTAACCTATGGGCTGTG |
| *Atwapl1-1*-genotyping-F | CGACGCACTTTCCGTCC |
| *Atwapl1-1*-genotyping-R | GAGCCAACGGTCGAGTA |
| *Atwapl2*-genotyping-F | GGTCTCAACAGCTTAACC |
| *Atwapl2*-genotyping-R | GAGCGAACTTACGGCCGTCG |
| *Atswi1*-genotyping-F | ACTCATCACCGCTTGATTCTG |
| *Atswi1*-genotyping-R | TGATACTGCACACGCAATCTC |
| HsBHC80PHD_486-543_-BamHI-F | CGCGGATCCATTCATGAGGATTTTTGCAGC |
| HsBHC80PHD_486-543_-SalI-R | ACGCGTCGACTTAAATTGCTTCTTCCTTCTTCAG |
| AtING2PHD-BamHI-F | CGCGGATCCTACTGTGTCTGCCATCAG |
| AtING2PHD-EcoRI-R | CCGGAATTCTTATCTGCAGGTGGGGCAGTAC |
| AtSCC2PHD_701-750_-BamHI-F | CGGGATCCGATTGCACATTTTGTTTAGGTAAAAG |
| AtSCC2PHD_701-750_-SalI-R | ACGCGTCGACTTATTGCCTCTTGCATACACAAAG |
| AtSCC2PHD_687-775_-BamHI-F | CGGGATCCGAGTCAGATAGTGAAATTAG |
| AtSCC2PHD_687-775_-SalI-R | GCGTCGACTTATTCGATGCTTTCTTCTGATTC |
| *AtSCC4*-RT-P1-F | TTCCTGAGCTCCAGATGTTC |
| *AtSCC4*-RT-P1-R | GGGCAGCGATCAGTCTTGTC |
| *AtSCC4*-RT-P2-F | AGCTTCTGAGTGTATGATTG |
| *AtSCC4*-RT-P2-R | TCTCTGTCAACTTGGTCGCTTC |
| *AtSCC4*-RT-P3-F | TTGCACGACACCGTACAAGC |
| *AtSCC4*-RT-P3-R | GCTGATACAGAGCTGTGAAG |
| AD-AtSCC2_1-254_-NdeI-recF | GTACCAGATTACGCTCATATGATGAGCAATCCAAGCAGTTCCG |
| AD-AtSCC2_1-254_-BamHI-recR | CAGCTCGAGCTCGATGGATCCTTAGCCACAGAAGTCCTCCAACATC |
| AD-AtSCC2_255-427_-NdeI-recF | GTACCAGATTACGCTCATATGAGAGCTGAAGTCCCTGGTGATG |
| AD-AtSCC2_255-427_-BamHI-recR | CAGCTCGAGCTCGATGGATCCTTATGACGCCTTCTTTACTTTGC |
| BD-AtSCC4-NdeI-recF | TCAGAGGAGGACCTGCATATGATGGAAGGTGCTGCTGTGGC |
| BD-AtSCC4-BamHI-recR | CCGCTGCAGGTCGACGGATCCTTACATCCTTCGTTTTCCCCATC |
| pXY103-104-AtSCC2-recF | ATTACAGGTACCCGGGGATCCATGAGCAATCCAAGCAGTTCCG |
| pXY104-AtSCC2quTAA-recR | CACGCTGCCACCGCCGTCGACCCTTCTGTTGCTACTTCTGGTG |
| pXY106-AtSCC4recF | ATCGAGGACGCCGGCGGATCCATGGAAGGTGCTGCTGTGGC |
| pXY105-106-AtSCC4jiaTAA-recR | ACGAAAGCTCTGCAGGTCGACTTACATCCTTCGTTTTCCCCATC |
